# Distant coupling between RNA editing and alternative splicing of the osmosensitive cation channel Tmem63b

**DOI:** 10.1074/jbc.RA120.016049

**Published:** 2021-01-13

**Authors:** Dan Wu, Yan-Yu Zang, Yong-Yun Shi, Chang Ye, Wen-Min Cai, Xiao-Hui Tang, Liyun Zhao, Yong Liu, Zhenji Gan, Gui-quan Chen, Yun Xu, Jian-Jun Yang, Yun Stone Shi

**Affiliations:** 1 Ministry of Education Key Laboratory of Model Animal for Disease Study, Model Animal Research Center, Medical School, Nanjing University, Nanjing, China; 2 State Key Laboratory of Pharmaceutical Biotechnology, Department of Neurology, Affiliated Drum Tower Hospital of Nanjing University Medical School, Nanjing University, Nanjing, China; 3 Department of Orthopaedics, Luhe People's Hospital Affiliated to Yangzhou University, Nanjing, China; 4 Department of Anesthesiology and Perioperative Medicine, First Affiliated Hospital of Zhengzhou University, Zhengzhou, China; 5 School of Medicine, Southeast University, Nanjing, China; 6 Translational Science and Clinical Biomarker, BeiGene (Shanghai) Co., Ltd., Shanghai, China; 7 Hubei Key Laboratory of Cell Homeostasis, College of Life Sciences, Institute for Advanced Studies, Wuhan University, Wuhan, China; 8 Institute for Brain Sciences, Nanjing University, Nanjing, China; 9 Chemistry and Biomedicine Innovation Center, Nanjing University, Nanjing, China

**Keywords:** Tmem63b, A-to-I RNA editing, alternative splicing, brain-specific, mechanosensitive, osmosensitive, mechanotransduction, RNA editing, osmotic swelling, ion channel

## Abstract

Post-transcriptional modifications of pre-mRNAs expand the diversity of proteomes in higher eukaryotes. In the brain, these modifications diversify the functional output of many critical neuronal signal molecules. In this study, we identified a brain-specific A-to-I RNA editing that changed glutamine to arginine (Q/R) at exon 20 and an alternative splicing of exon 4 in *Tmem63b*, which encodes a ubiquitously expressed osmosensitive cation channel. The channel isoforms lacking exon 4 occurred in ∼80% of *Tmem63b* mRNAs in the brain but were not detected in other tissues, suggesting a brain-specific splicing. We found that the Q/R editing was catalyzed by Adar2 (Adarb1) and required an editing site complementary sequence located in the proximal 5′ end of intron 20. Moreover, the Q/R editing was almost exclusively identified in the splicing isoform lacking exon 4, indicating a coupling between the editing and the splicing. Elimination of the Q/R editing in brain-specific *Adar2* knockout mice did not affect the splicing efficiency of exon 4. Furthermore, transfection with the splicing isoform containing exon 4 suppressed the Q/R editing in primary cultured cerebellar granule neurons. Thus, our study revealed a coupling between an RNA editing and a distant alternative splicing in the *Tmem63b* pre-mRNA, in which the splicing plays a dominant role. Finally, physiological analysis showed that the splicing and the editing coordinately regulate Ca^2+^ permeability and osmosensitivity of channel proteins, which may contribute to their functions in the brain.

RNA editing is a post-transcriptional modification of pre-mRNAs that can introduce codon changes in mature mRNAs. The A (adenosine)-to-I (inosine) deamination in pre-mRNAs is the most abundant RNA editing in mammals (1). Because inosine in mRNA is interpreted as guanosine (G) during translation (2), the A-to-I editing often leads to changes in amino acid sequence. The A-to-I editing occurs in important signal molecules, including α-amino-3-hydroxy-5-methyl-4-isoxazolepropionic acid receptor GluA2 subunit (3), kainate receptor GluK1 and GluK2 subunits (4), Kv1.1 α subunit, and 5-HT2C receptors (5, 6), which are known to regulate neuronal development, circuit formation, neuronal degeneration, and synaptic transmission (7, 8, 9, 10). The A-to-I editing is catalyzed by Adar (adenosine deaminases acting on RNA) enzymes, which recognize the dsRNA hairpin structure formed by the editing region and the editing site complementary sequence (ECS) and deaminate the targeted adenosine to inosine (11). In mammals, Adar1 (Adar) and Adar2 (Adarb1) catalyze deamination, whereas Adar3 (Adarb2) has no enzyme activity (1).

Alternative splicing is another type of post-transcriptional modification of pre-mRNAs in eukaryotes (12). Whereas more than 95% of genes undergo alternative splicing in human, around 63% do so in mouse (13, 14). Aberrant alternative splicing is widely observed in various diseases including Mediterranean anemia (15), Alzheimer's disease (16, 17), spinal muscular atrophy (18), ALS (19, 20), and cancers (21). Intriguingly, the A-to-I RNA editing in exons and the alternative splicing of nearby introns are often coupled. For instance, the R/G editing at exon 13 of *Gria2* regulates alternative splicing of exon 14 (flop) and exon 15 (flip) (22, 23, 24). The splicing efficiency also regulates nearby upstream editing when it is guided by intronic ECS (25, 26).

We have recently found that Tmem63b serves as an osmosensor in the inner ear and is required for survival of outer hair cells and hearing (27). To expand our understanding of Tmem63b functions in other systems, we cloned *Tmem63b* mRNA from mouse brain in the current study. We identified four isoforms of *Tmem63b* resulting from an A-to-I RNA editing that changes glutamine to arginine at exon 20 and an alternative splicing of exon 4. The editing was almost exclusively detected in the spliced isoform lacking exon 4, suggesting a linkage between the two post-transcriptional events. Using *Adar2* cKO mice and cultured cerebellar granule neurons (CGNs), we found that the isoform containing exon 4 suppressed the Q/R editing efficiency in a *cis* manner. Functional analysis demonstrated that the splicing and the editing coordinately regulated hypoosmolarity-induced Ca^2+^ influx. Together, these results reveal a long-distance coupling between alternative RNA splicing and RNA editing in *Tmem63b*, in which the splicing plays a dominant role. These post-transcriptional modifications may enable the osmosensitive Tmem63b channel to play diversified roles in the brain.

## Results

### Q/R editing at exon 20 and alternative splicing of exon 4 in Tmem63b cDNAs

**Figure 1:**
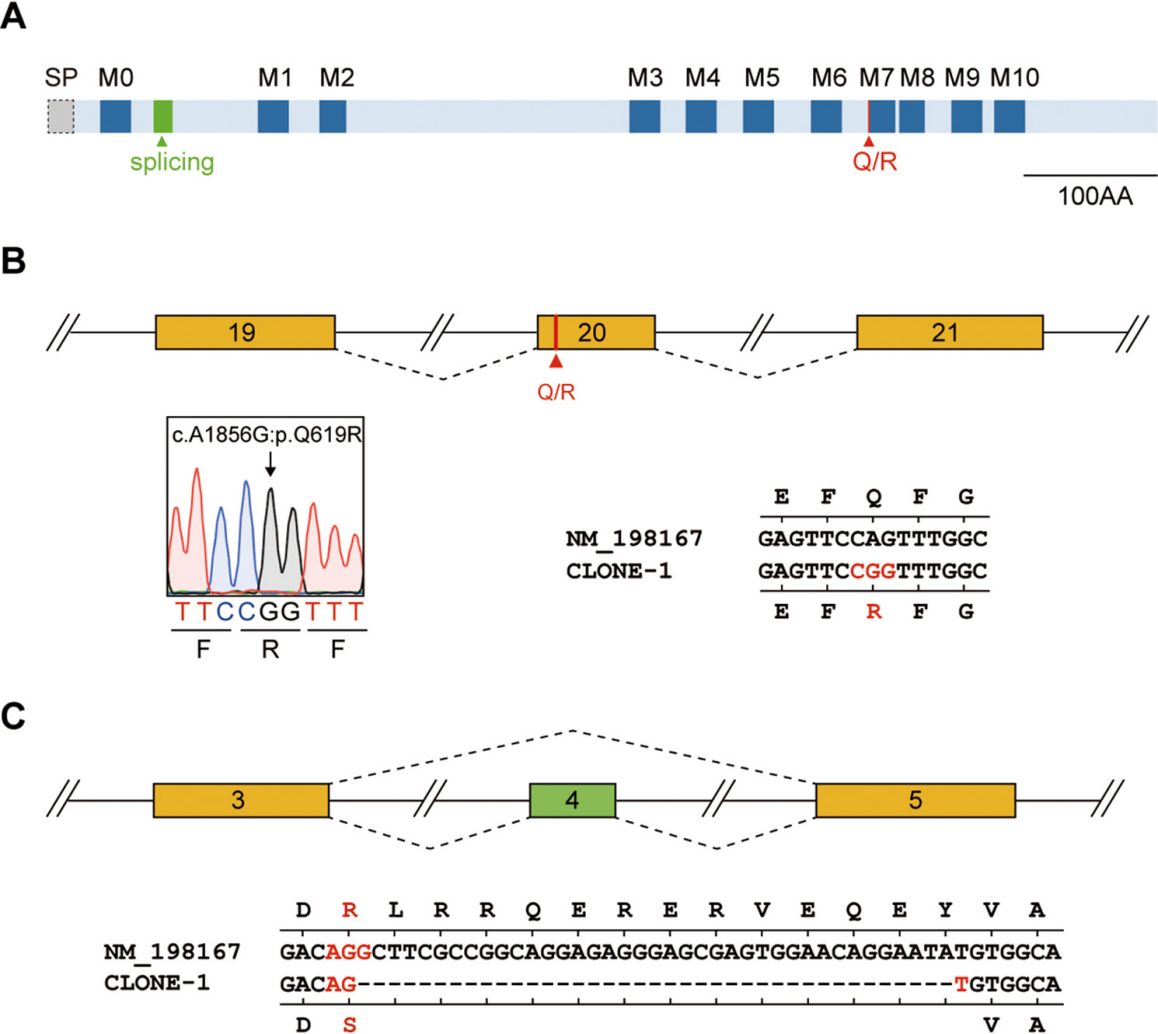
**Q/R editing site at exon 20 and alternative splicing of exon 4 in *Tmem63b* pre-mRN*A*.***A*, domain structure of the mouse Tmem63b protein. The transmembrane domains (M0 to M10) are depicted in *blue* with linking regions in *light blue*. The A-to-I editing (*red line*) is at the intracellular linker adjacent to M7. The alternative splicing exon 4 (*green block*) encodes amino acids in the first intracellular loop. *SP*, signal peptide. *Scale bar*, the size of the *line diagram*. *B*, schematic representation of Q/R editing site at exon 20. *Lower-left* shows the sequence of the Q/R site cloned from mouse brain cDNA. *Lower-right* shows the cloned sequence (CLONE-1) compared with documented *Tmem63b* mRNA sequence NM_198167. The corresponding amino acids are shown above and under the nucleotide sequences. *C*, schematic representation of *Tmem63b* exons 3–5. The comparison between cloned sequence (CLONE-1) and documented *Tmem63b* sequence is shown below. The corresponding amino acids are shown above and under the nucleotide sequences.

Recently, the osmosensitive cation channels in TMEM63 family have attracted research interests (27, 28, 29). When analyzing *Tmem63b* cDNAs from mouse brain tissues, we obtained one cDNA sequence that was different from the documented mRNA sequence NM_198167 in the NCBI database (Fig. 1*A*). The nucleotide c.1856 in this cDNA was guanosine, instead of the published adenine at the same site in exon 20. This finding suggested an A-to-I RNA editing that results in the substitution of glutamine codon CAG to arginine codon CGG at position 619 of the protein sequence (Fig. 1*B*). In addition, exon 4 was spliced out in the cDNA (Fig. 1*C*). Thus, the *Tmem63b* transcript we obtained was a new isoform with two post-transcriptional modifications, *i.e.* A-to-I editing at c.1856 in exon 20 and alternative splicing of exon 4.

### Q/R editing of Tmem63b varies with brain regions and ages

**Figure 2:**
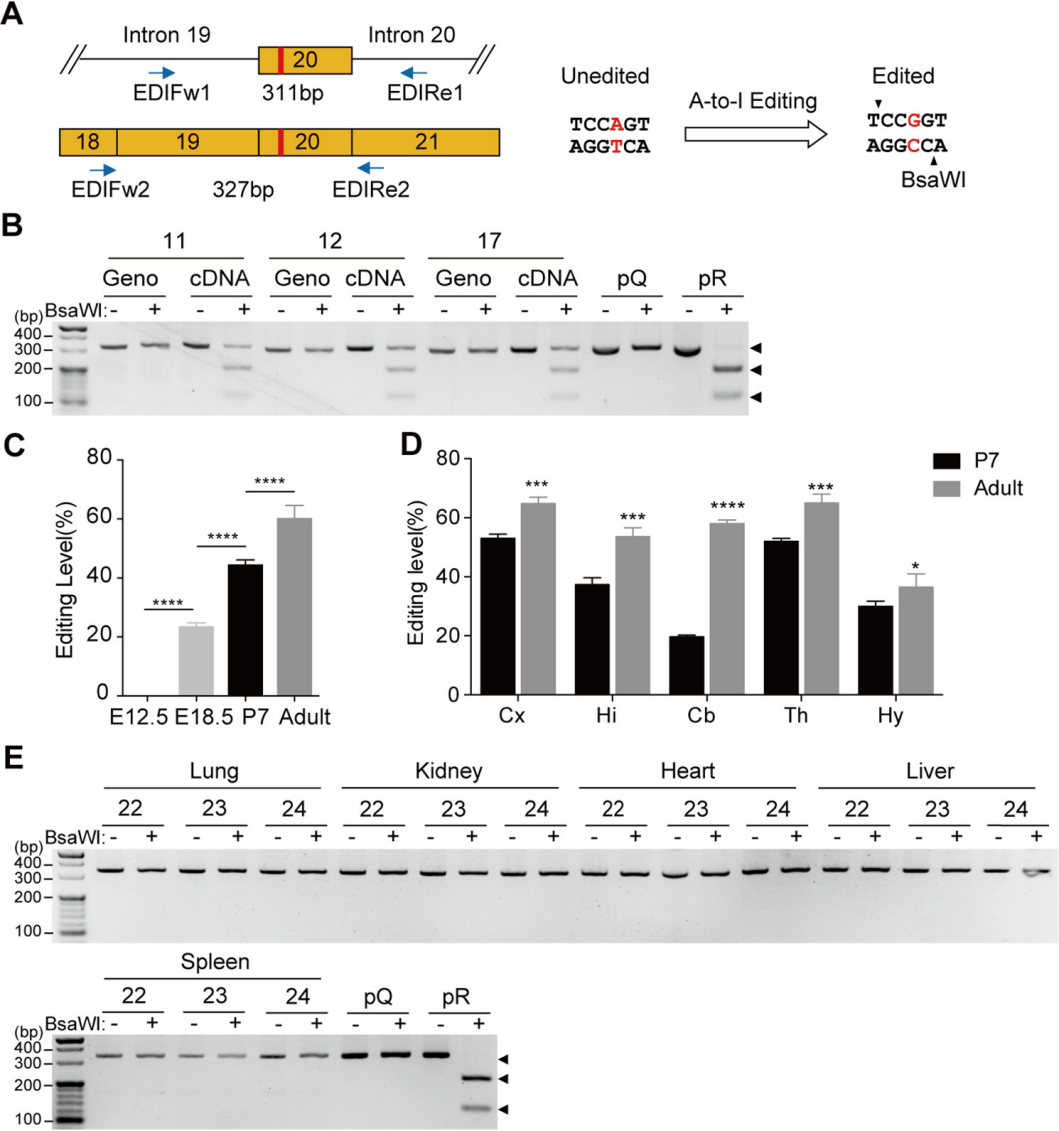
**The brain-specific Q/R editing of *Tmem63b* varies with brain regions and ages.***A*, schematic demonstration of genomic (*upper panel*) and cDNA (*lower panel*) sequence around *Tmem63b* exon 20 with Q/R editing site marked in *red*. Primers for amplification are indicated. Q/R editing led to a BsaWI endonuclease recognizing site (*right panel*). *B*, Q/R editing of *Tmem63b* in brains of three adult mice with codes labeled above. The amplified sequences with unedited (Q) and edited (R) forms were indicated by *black arrowheads*. Plasmids with Q-form (*pQ*) and R-form (*pR*) *Tmem63b* cDNAs served as control to indicate the enzymatic activity of BsaWI. *C*, quantification of Q/R editing levels in brains of E12.5 (0.0 ± 0.0%, *n* = 3), E18.5 (23.7 ± 1.2%, *n* = 3), P7 (44.6 ± 1.5%, *n* = 5), and adult (60.3 ± 4.2%; *n* = 6) mice. Data are shown as mean ± S.D. (*error bars*). ****p < 0.0001; one-way ANOVA, Tukey's post hoc test. *D*, quantification of Q/R editing levels in cerebral cortex (*Cx*), hippocampus (*Hi*), cerebellum (*Cb*), thalamus (*Th*), and hypothalamus (*Hy*) of P7 (*black column*) and adult (*gray column*) mice. Data are shown in Table S1 as mean ± S.D. *p < 0.05; ***p < 0.001; ****p < 0.0001; Student's *t* test, two-tailed. *E*, Q/R editing of *Tmem63b* was absent in lungs, kidneys, hearts, livers, and spleens from three adult mice with codes labeled above.

The conversion of A to G at the 1856 site creates an endonuclease site for BsaWI (Fig. 2*A*). Specific primer pairs were designed to amplify sequences containing the editing site from mouse genomic DNAs (311 bp) and brain cDNAs (327 bp) (Fig. 2*A*). The majority of PCR products amplified from brain cDNAs of adult mice were digested by BsaWI and showed two additional bands (208 bp and 119 bp), whereas the PCR products amplified from mouse genomic DNAs were not recognized by BsaWI (Fig. 2*B*). BsaWI failed to digest *Tmem63b* cDNA fragments from tissues other than brain, including lung, kidney, heart, liver, and spleen (Fig. 2*E*), suggesting that this editing is brain-specific. In addition, the editing efficiency varied in brain sub-regions, from ∼40% in the hypothalamus to ∼60% in other brain regions in adult mice (Fig. 2*D* and Fig. S1, A and C). To investigate whether such editing was affected during the development, we examined the Q/R editing in P7 and embryonic (E12.5 and E18.5) brains and observed lower editing efficiency as compared with the adult brains (Fig. 2*C* and Fig. S1, B and D). There was virtually no editing in the brains of E12.5 mice, likely because of low expression level of Adar enzymes at this stage (1). Results from different brain regions at P7 were similarly lower than those in the adult brain (Fig. 2*D* and Fig. S1, A and C).

### Q/R editing of Tmem63b is catalyzed by Adar2

**Figure 3:**
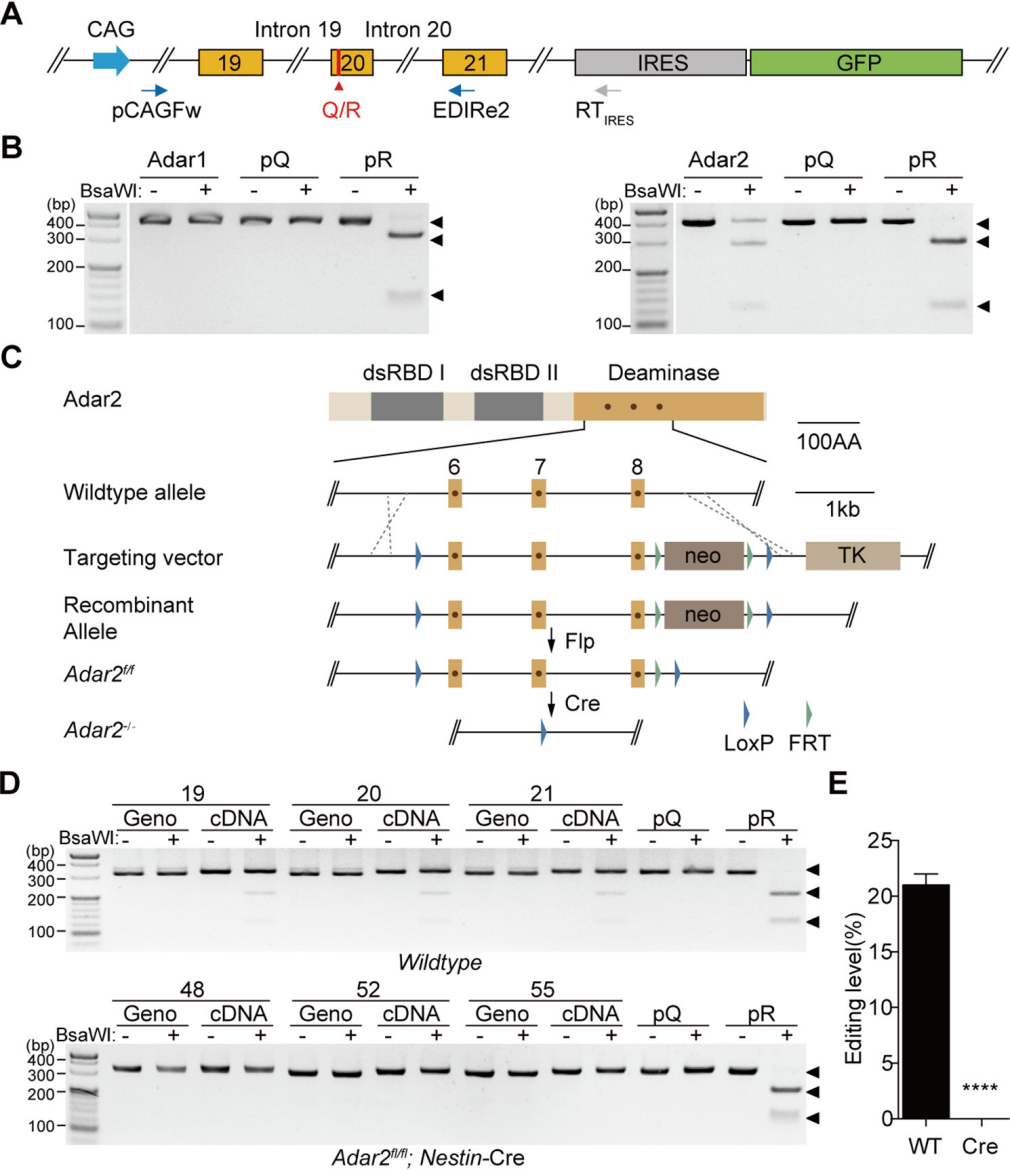
**Q/R editing of *Tmem63b* is catalyzed by Adar2.***A*, schematic representation of the minigene construct containing the editing segment of *Tmem63b*. The minigene was driven by CAG promoter and linked with GFP through IRES. pCAGFw and EDIRe2 are primers for amplification. *B*, Q/R editing of *Tmem63b* minigene in HEK293 cells expressing Adar1 or Adar2. *C*, the generation of *Adar2* floxed (*Adar2^fl/fl^*) mice by homologous recombination techniques. The *dots* within the targeted exons indicate the three amino acid residues essential for the deaminase activity. *dsRBD*, dsRNA binding domains. *TK*, thymidine kinase. *neo*, neomycin. *D*, Q/R editing in brains from *WT* and brain-specific *Adar2* knockout P0 mice. The experiments were repeated in three *WT* mice (labeled as 19, 20, and 21) and three *Adar2* cKO mice (labeled as 48, 52, and 55). *E*, quantification of the Q/R editing levels in brains from *WT* mice (21.0 ± 1.0%, *n* = 3) and brain-specific *Adar2* knockout mice (0.0 ± 0.0%, *n* = 3). Data are shown as mean ± S.D. (*error bars*). ****p < 0.0001; Student's *t* test, two-tailed. *FRT*, Flp recognition target.

In mammals, Adar1 (Adar) and Adar2 (Adarb1) catalyze A-to-I editing. To determine whether Adar1 or Adar2 catalyzed the Q/R editing in *Tmem63b*, we transfected mouse Adar1 or Adar2 cDNA together with a *Tmem63b* minigene containing the genomic sequence from exon 19 to exon 21 (the editing segment) in HEK293 cells and evaluated the editing efficiency (Fig. 3*A*). To avoid putative contamination from endogenous *Tmem63b* in HEK cells, the exogenous RNAs were reverse-transcribed into cDNAs by a primer RT_IRES (Internal Ribosome Entry Site)_ paired to vector sequence (Fig. 3*A*). Adar2 but not Adar1 transfection resulted in partial digestion of PCR products (403 bp) into two bands (284 bp and 119 bp) by BsaWI (Fig. 3*B*). To verify that the transfected Adar1 was functional, we coexpressed Adar1 with an *Htr2c* minigene containing the “A” editing site at exon 5 that is preferentially edited by Adar1 (30) and found that this site was efficiently edited (Fig. S2). To examine whether the *Tmem63b* Q/R editing was catalyzed by Adar2 *in vivo*, we generated *Adar2* floxed mice (*Adar2^fl/fl^*) and bred them with *Nestin-cre* line (31) to obtain brain-specific *Adar2* cKO (*Adar2^fl/fl^;Nestin-Cre*) mice (Fig. 3*C*). The *Adar2* cKO mice were postnatally lethal, likely because of inability to edit the *Gria2* Q/R site (32). We therefore analyzed the Q/R editing of *Tmem63b* in P0 mouse brains. The Q/R editing of *Tmem63b* was completely eliminated in *Adar2* cKO brains (Fig. 3, *D* and *E*).

### Analysis of the downstream ECS

**Figure 4:**
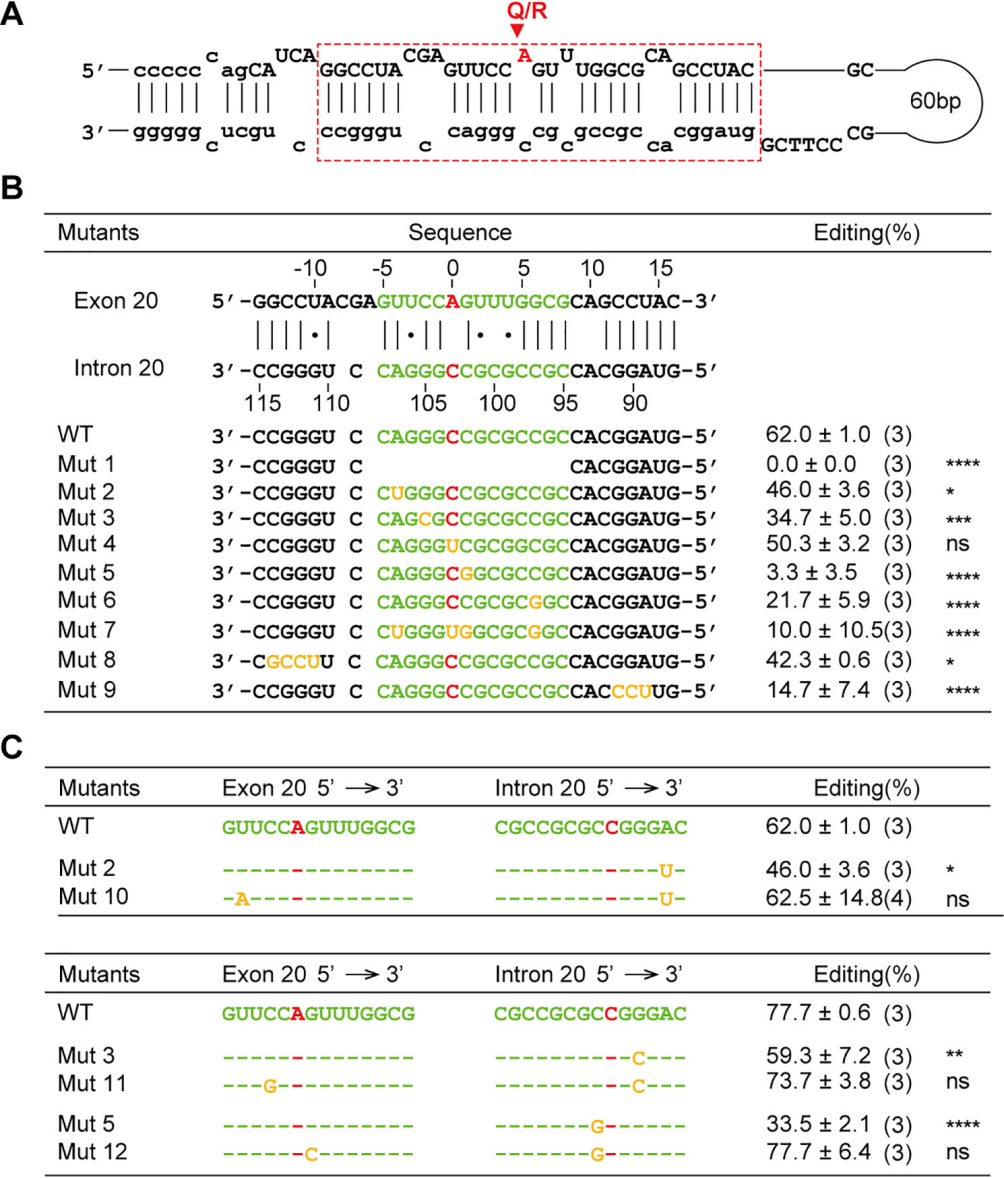
**Analysis of downstream ECS essential for Q/R Editing.***A*, schematic representation of dsRNA hairpin structure formed by the editing region and the ECS, with the ECS marked in *red frame* and Q/R editing site in *red arrowhead*. *B*, Q/R editing of *Tmem63b* minigenes with mutations on ECS. The extent of Q/R editing was determined as illustrated in Fig. 3*A*. *C*, compensatory mutations in the editing region rescue editing efficiency impaired by mutations in ECS. The editing efficiency was quantified by measuring the peak heights ratio at the Q/R site obtained from sequencing chromatograms in the *lower table*. Data are shown as mean ± S.D. *p < 0.05; **p < 0.01; ***p < 0.001; ****p < 0.0001; *ns*, not significant; one-way ANOVA, Tukey's post hoc test. The sample numbers of replicates are listed in parentheses.

To dissect the critical ECS for Q/R editing of *Tmem63b*, we analyzed the secondary structure of RNA sequence surrounding editing site using the bioinformatics software RNAstructure (33). A dsRNA duplex containing Q/R editing site was obtained, and the sequence base-paired to the editing region was predicted as ECS (in *red frame*, Fig. 4*A*). We then introduced a series of mutations on the predicted ECS and analyzed their effects on Q/R editing in Adar2 coexpressed HEK 293 cells. The editing efficiency in WT sequence was 62%, whereas deletion of 14 consecutive nucleotides from the center of ECS abolished Q/R editing (*Mut 1*, Fig. 4*B*). In addition, mutations on single or multiple nucleotides that destabilized the dsRNA duplex also decreased the editing efficiency (*Mut 2–Mut 9*, Fig. 4*B*), whereas introduction of the compensatory mutations around the editing site restored the editing efficiency (*Mut 10–Mut 12*, Fig. 4*C*), indicating the importance of the hairpin structure formed by the intronic ECS and the exonic editing region. Taken together, the intronic sequence at the proximal 5′ end of intron 20 was demonstrated to be the ECS for *Tmem63B* Q/R editing.

### Alternative splicing of exon 4 is brain-specific

**Figure 5:**
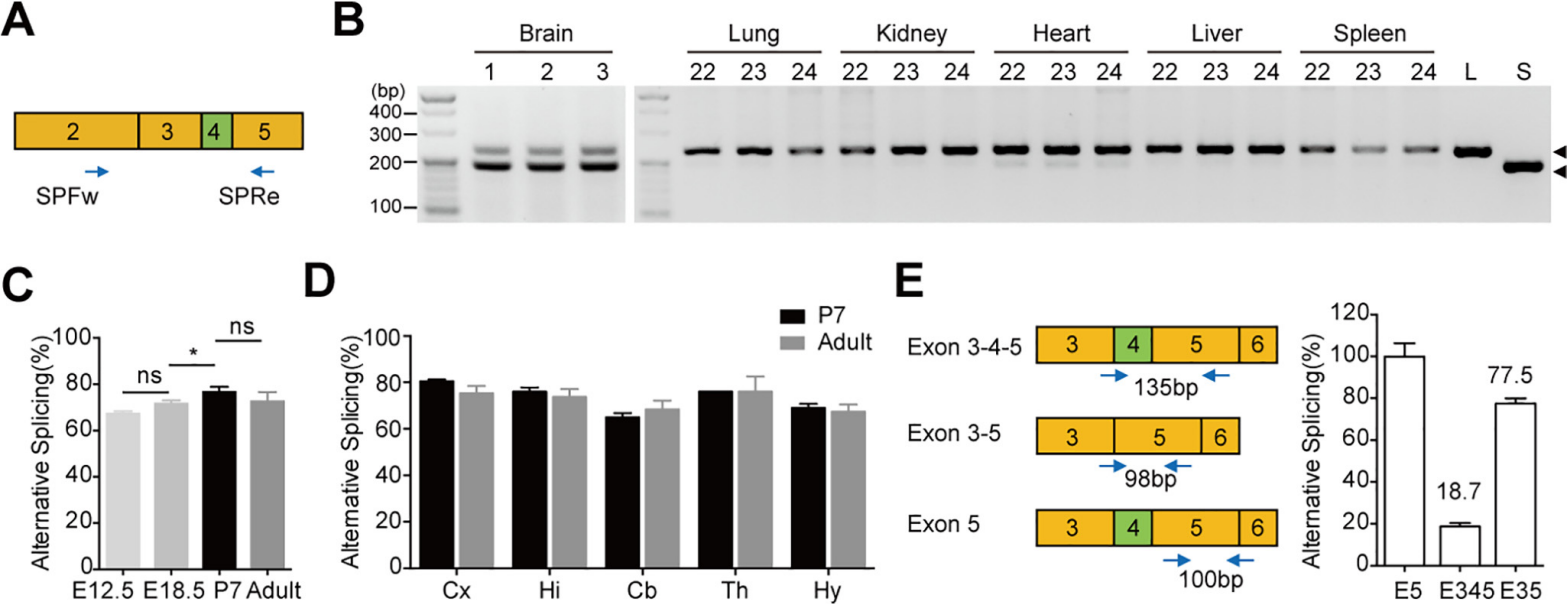
**Alternative splicing of *Tmem63b* exon 4 in mouse brains.***A*, schematic representation of *Tmem63b* exons 3–5 in cDNA with exon 4 marked in *green*. *SPFw* and *SPRe* are primers for amplification. *B*, alternative splicing of *Tmem63b* exon 4 in brains, lungs, kidneys, hearts, livers, and spleens from three adult mice with codes labeled above. The amplified sequences with long and short forms were indicated by *black arrowheads*. *C*, quantification of alternative splicing levels in brains of E12.5 (67.7 ± 0.6%, *n* = 3), E18.5 (72.0 ± 1.0%, *n* = 3), P7 (77.0 ± 1.9%, *n* = 5), and adult (73.0 ± 3.6%, *n* = 3) mice. Data are shown as mean ± S.D. (*error bars*) *p < 0.05; *ns*, not significant; one-way ANOVA, Tukey's post hoc test. *D*, quantification of alternative splicing levels in cerebral cortex (*Cx*), hippocampus (*Hi*), cerebellum (*Cb*), thalamus (*Th*), and hypothalamus (*Hy*) of P7 (*black column*) and adult (*gray column*) mice. Data are presented in Table S2 as mean ± S.D. *E*, *left*, schematic representation of *Tmem63b* cDNA sequence from exon 3 to exon 6. Primers for amplification are indicated. *Right*, quantification of alternative splicing levels in brains of P7 mice by real-time PCR, which were similar to the results of agarose gel electrophoresis. *E5*, exon 5 (*n* = 3); *E345*, exon 3-4-5 without intron interval (18.7 ± 1.6%, *n* = 3); *E35*, exon 3-5 without intron interval (77.5 ± 2.6%, *n* = 3).

To study alternative splicing of exon 4, a pair of primers, SPFw and SPRe, were designed to amplify the sequence around exon 4 (Fig. 5*A*). *Tmem63b* cDNAs with (long-form) or without (short-form) exon 4 were amplified into PCR products of different lengths, 223 bp and 184 bp, respectively. The efficiency of the splicing was calculated as the percentage of short form to total *Tmem63b*. The short form was detected in brain of adult mouse but not in lung, kidney, liver, and spleen (Fig. 5*B*). Notably, a weak splicing occurred in about 2% of total heart Tmem63b mRNAs. In the whole brains, ∼80% of the *Tmem63b* mRNAs were the short form. The splicing efficiency in different brain regions mildly varied from 70 to 80% (Fig. 5*D* and Fig. S3, A and C). In addition, the splicing efficiency in P7 mouse brains was similar to that in the adult brains, but it was modestly lower in the embryonic brains (E12.5 and E18.5), with the short form accounting for 68% of *Tmem63b* mRNAs at E12.5. These data demonstrated that the splicing efficiency in *Tmem63b* pre-mRNA was relatively stable during development (Fig. 5, *B* and *C* and Fig. S3, B and D).

Above data suggested that up to ∼80% of *Tmem63b* mRNAs from mouse brain were short isoforms lacking exon 4. One concern was that the PCR reaction using this pair of primers could prefer one isoform over the other. To perform an independent evaluation on the splicing efficiency, we designed three pairs of primers to amplify the short form, the long form, and the total *Tmem63b* mRNAs, respectively, and examined the splicing by quantitative real-time PCR (Fig. 5*E*). The results showed that the long form (exon 3-4-5) and the short form (exon 3-5) were 18.7% and 77.5%, respectively, of the total *Tmem63b* mRNAs (exon 5) (Fig. 5*E*). This experiment further verified that around 80% of mRNAs from adult mouse brain were short isoforms. Taken together, the alternative splicing of *Tmem63b* exon 4 is brain-specific. The splicing efficiency is constant among different brain regions and relatively stable at different development stages.

### Distant coupling between alternative splicing and editing in Tmem63b

**Figure 6:**
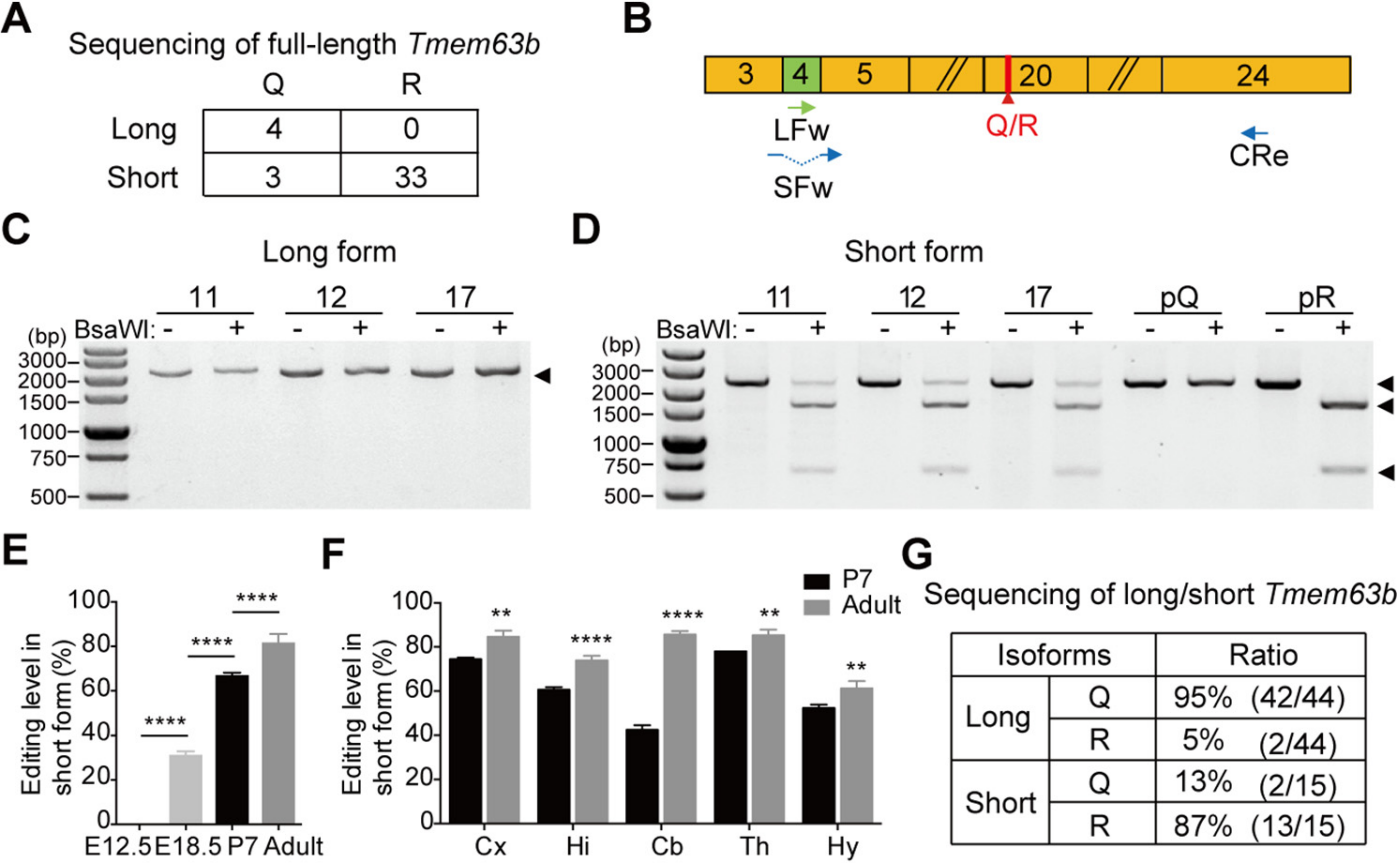
**The coupling between exon 4 alternative splicing and Q/R editing in *Tmem63b* pre-mRNA.***A*, summary of the sequencing results of full-length *Tmem63b* cDNAs from mouse brain. *B*, schematic representation of *Tmem63b* exons 3–24 in cDNA. Primers for amplification are indicated. *C* and *D*, Q/R editing of long-form and short-form *Tmem63b* in brains from three adult mice with codes labeled above. The amplified sequences with long and short forms are indicated by *black arrowheads*. Plasmids with Q-form (*pQ*) and R-form (*pR*) *Tmem63b* cDNAs served as control to indicate the enzymatic activity of BsaWI. *E*, quantification of Q/R editing levels of short-form *Tmem63b* in brains of E12.5 (0.0 ± 0%, *n* = 3), E18.5 (31.3 ± 1.5%, *n* = 3), P7 (67.0 ± 1.2%, *n* = 5), and adult (81.7 ± 3.9%, *n* = 6) mice. Data are shown as mean ± S.D. (*error bars*). ****p < 0.0001; one-way ANOVA, Tukey's post hoc test. *F*, quantification of Q/R editing levels of short-form *Tmem63b* in cerebral cortex (*Cx*), hippocampus (*Hi*), cerebellum (*Cb*), thalamus (*Th*), and hypothalamus (*Hy*) of P7 (*black column*) and adult (*gray column*) mice. Data are presented in Table S3 as mean ± S.D. **p < 0.01; ****p < 0.0001; Student's *t* test, two-tailed. *G*, summary of the sequencing results of long-form and short-form *Tmem63b* from mouse brains.

Previous studies demonstrated that A-to-I editing in exons and splicing of nearby downstream introns could affect each other (22, 23, 24, 25, 26). To test whether the Q/R editing at exon 20 and the splicing of exon 4 in *Tmem63b* pre-mRNA are also coupled, 40 *Tmem63b* clones originated from mouse brain were subjected to Sanger sequencing with full length, of which 36 were short cDNA clones lacking exon 4 and the majority of them (33/36) contained the edited R at the Q/R site. In contrast, all four long cDNA clones were not edited at the Q/R site (Fig. 6*A*). These data suggested that Q/R editing efficiency in long and short isoforms was biased. We further analyzed this phenotype using BsaWI digestion. A pair of primers, with the forward primer base-paired to exon 4 (LFw) and the reverse one downstream of the Q/R site (CRe), was used to amplify the long isoform (2255 bp). The forward primer, with 4 bp of the 3′ end base-paired to exon 5 and the rest of sequence base-paired to exon 3 (SFw), was designed to amplify the short isoform (2246 bp) (Fig. 6*B*). We found that the fragments amplified from short isoform were largely digested by BsaWI (1593 bp and 653 bp) whereas the digestion bands were not seen in long-form *Tmem63b* fragments (Fig. 6, *C* and *D*). The Q/R editing was also limited to the short-form mRNAs of P7 and E18.5 mouse brains (Fig. S4, B and D), indicating that coupling is also present during development. As expected, the editing efficiency in short form was lower in young brains (Fig. 6*E*). The editing efficiency in short-form *Tmem63b* was further analyzed in different brain regions of P7 and adult mice. In adult, the editing was ∼60% in hypothalamus and ∼80% in other brain regions, whereas in P7, the editing in short-form mRNAs was generally lower in all brain regions as compared with those in adult (Fig. 6*F* and Fig. S4, A and C). To test whether Q/R editing is mutually excluded from the long-form *Tmem63b*, we subcloned the above fragments amplified from long- or short-form *Tmem63b* into pMD19-T vectors and subjected them to Sanger sequencing. In 44 clones of the long isoform, 42 contained unedited Q and two clones were edited at the Q/R site. In contrast, 87% (13/15) of the clones amplified from the short isoform were edited to R at the Q/R site (Fig. 6*G*). Taken together, these results indicated that the alternative splicing of exon 4 and Q/R editing at exon 20 in *Tmem63b* pre-mRNA were coupled.

### Presence of exon 4 suppresses Q/R editing in Tmem63b

**Figure 7:**
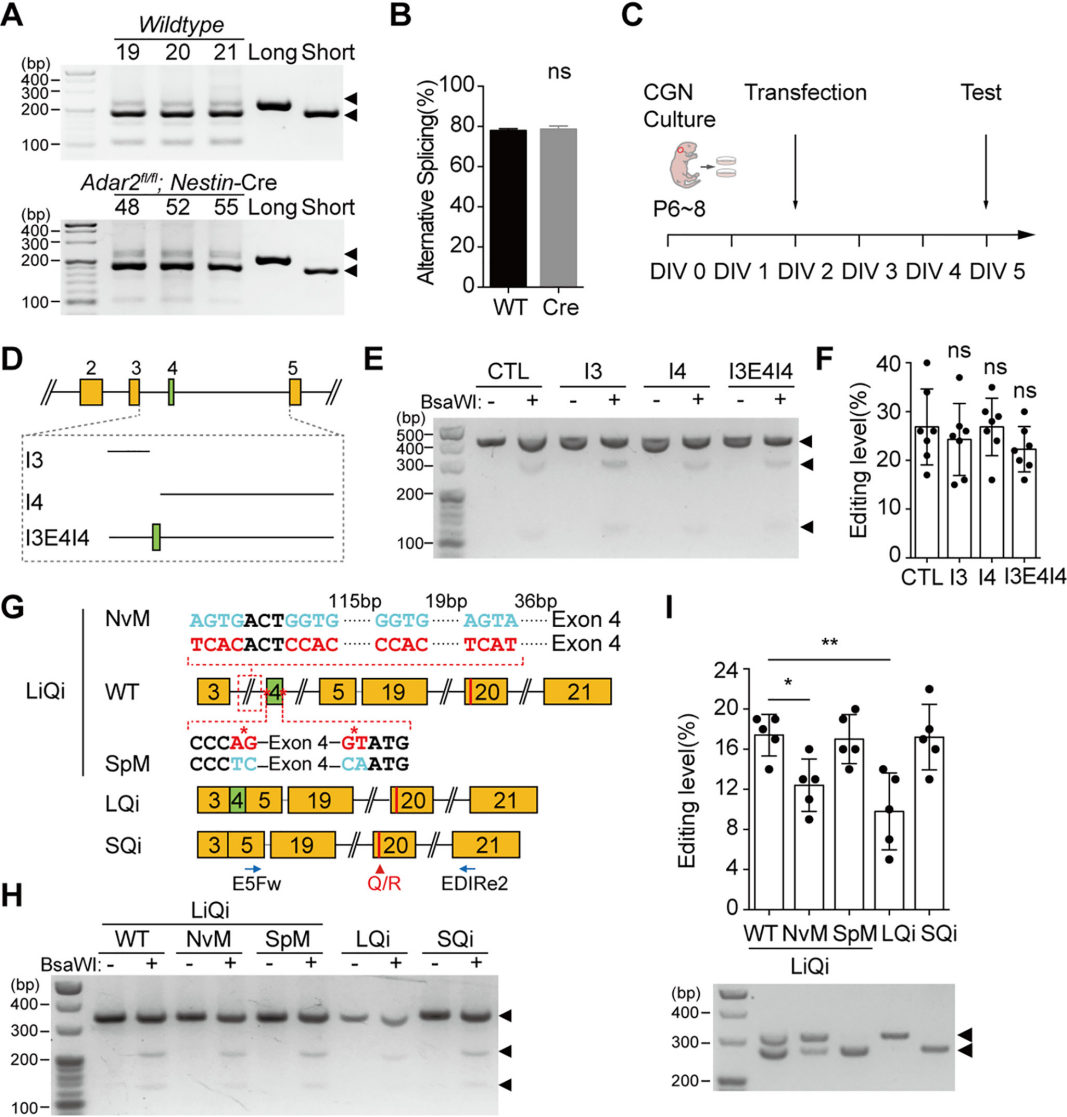
**Alternative splicing of exon 4 regulates Q/R editing at exon 20.***A*, alternative splicing of exon 4 in brains of *WT* and *Adar2* cKO mice. *B*, quantification of alternative splicing levels in brains of *WT* (78.0 ± 1.0%, *n* = 3) and *Adar2* cKO (78.7 ± 1.5%, *n* = 3) mice. Data are shown as mean ± S.D. *ns*, not significant; Student's *t* test, two-tailed. *C*, schematic demonstration of the experiments conducted in CGNs. *D*, schematic demonstration of the intronic segments spliced out in alternative splicing. *E* and *F*, Q/R editing efficiencies of the editing segment when cotransfected with I3 (24.3 ± 7.4%, *n* = 7), I4 (26.9 ± 5.9%, *n* = 7), I3E4I4 (22.3 ± 4.6%, *n* = 7), and vehicle control (*CTL*) (26.9 ± 7.8%, *n* = 7) in CGNs. Data are shown as mean ± S.D. (*error bars*). *ns*, not significant; one-way ANOVA, Tukey's post hoc test. *G*, schematic demonstration of WT and mutant minigene constructs fuzing the editing segment and splicing segment. E5Fw and EDIRe2 are primers for amplification. *H* and *I*, Q/R editing of fusion minigenes in CGNs. The Q/R editing efficiency was not changed in SQi (17.2 ± 3.3%, *n* = 5) and SpM (17.0 ± 2.4%, *n* = 5) but was suppressed in LQi (9.8 ± 3.8%, *n* = 5) and NvM (12.4 ± 2.6%, *n* = 5) mutant, compared with the LiQi control (17.4 ± 2.1%, *n* = 5). *Lower panel* in (*H*) shows the splicing levels corresponding to the upper editing event. Data are shown as mean ± S.D. (*error bars*). *p < 0.05; **p < 0.01; one-way ANOVA, Tukey's post hoc test.

We then studied which of the post-transcriptional modifications, the editing or the splicing, was dominant. We reasoned that if the editing determined the splicing efficiency as reported previously (22, 23, 24), then the alternative splicing of exon 4 would be absent in *Adar2* cKO mice. However, we found that the efficiency of alternative splicing was not changed (Fig. 7, *A* and *B*), suggesting that the editing did not affect the splicing.

To test the possibility that the splicing regulates the editing, we used primary culture of CGNs. We first examined the coupling of splicing and editing of endogenous *Tmem63b* in these neurons at DIV5, a time point at which we examined the transfected constructs (Fig. 7*C*). The splicing efficiency (75.7 ± 2.1%, *n* = 3, Fig. S5A) was close to that in the whole brain of P7 mice (Fig. 5*C*). The editing efficiency of endogenous *Tmem63b* in cultured CGNs was 28.7 ± 2.1% (*n* = 3, Fig. S5B), close to that in the P7 mouse cerebellum (∼20%, Fig. 2*D*). Importantly, the editing was only detected in short isoforms using BsaWI digestion, with editing efficiency of 37% (Fig. S5, C and D). We then examined the splicing and editing efficiencies of *Tmem63b* minigenes transfected in cultured CGNs. The editing segment of *Tmem63b* (Fig. 3*A*) was efficiently edited (26.9 ± 7.8%, *n* = 7) when transfected into CGNs (*CTL*, Fig. 7, *D–F*). The alternative splicing might modulate the editing through a *trans* mechanism, where the introns spliced out from long- or short-form *Tmem63b* regulate the editing, or a *cis* mechanism, where the mRNA sequence around the splicing region affects the editing. For the long-form *Tmem63b*, the introns spliced out are intron 3 (I3) and intron 4 (I4), whereas in short form, the intronic segment spliced out is I3E4I4 (Fig. 7*D*). We found that all these intronic segments had no effects on the editing efficiency (Fig. 7, *E* and *F*), arguing against a *trans* mechanism. Then we constructed minigenes by fusing the editing segment with DNA segments containing exon 3∼5 varied by alternative splicing of exon 4 (*LiQi*, *LQi and SQi*, Fig. 7*G*). The LiQi (genomic sequence from exon 3 to exon 5 linked with the editing segment) was largely (∼65%) spliced to short form (Fig. 7*I*, *lower panel*). The editing efficiency in LiQi (17.4 ± 2.1%, *n* = 5) was close to SQi (exon 3-5 linked with the editing segment, 17.2 ± 3.3%, *n* = 5) but higher than LQi (exon 3-4-5 linked with the editing segment, 9.8 ± 3.8%, *n* = 5), indicating that the splicing regulates the editing in a cis manner. We then wondered whether the editing efficiency can be modified by changing splicing. We first made splicing site mutant (SpM), 5′ splice site (GU) at intron 4 and 3′ splice site (AG) at intron 3 mutated to complimentary nucleotides, which disrupts the inclusion of exon 4 (Fig. 7*G*). As expected, only the short-form transcript was observed. The editing in SpM (17.0 ± 2.4%, *n* = 5) was the same as SQi (Fig. 7, *H* and *I*). In neurons, multiple splicing factors such as Nova (34), Rbfox (35), nSR100 (36), Ptbp1, and Ptbp2 (37) enhance or silence splicing events through binding to specific RNA motifs. There are Nova binding motifs (YCAY clusters) upstream of exon 4 (Fig. 7*G*) which could be involved in Nova-mediated exon skipping (38). These sites were then simultaneously mutated to obtain Nova binding motif mutant (NvM) (Fig. 7*G*). As expected, LiQi harboring these mutations significantly reduced the short isoform (∼40% in NvM *versus* ∼65% in WT LiQi). The editing efficiency was lower in NvM (12.4 ± 2.6%, *n* = 5) as compared with WT LiQi (Fig. 7, *H* and *I*). Taken together, these data indicate that the splicing of exon 4 regulated Q/R editing through a *cis* mechanism.

### The Q/R editing affects Ca^2+^ permeability of Tmem63b channel

**Figure 8:**
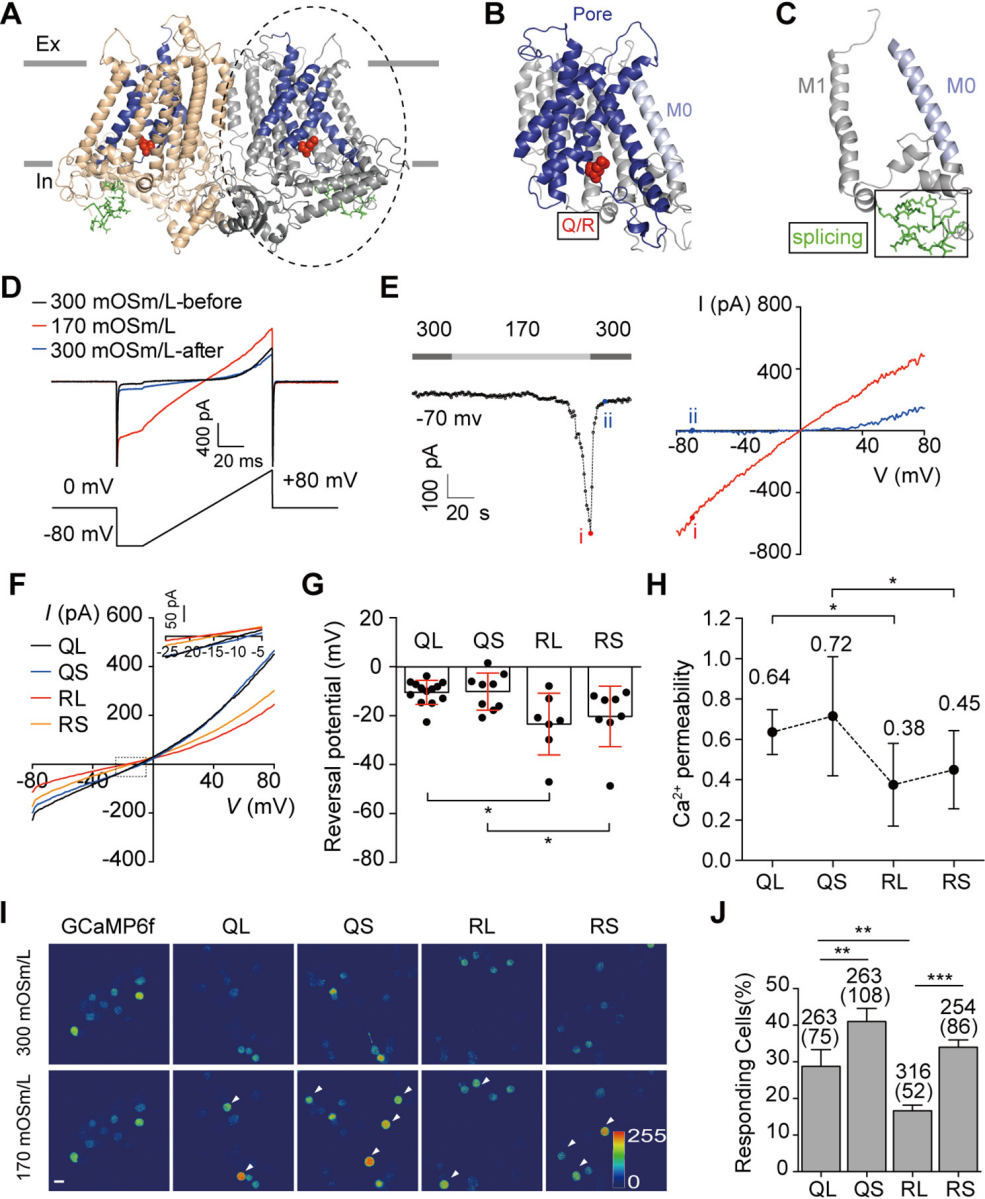
**The splicing and the editing in *Tmem63b* regulate Ca^2+^ influx in response to hypotonic stimuli.***A–C*, the 3-dimensional homology model of mouse Tmem63b based on *At*OSCA1.2 (PDB ID: 6mjv). The Q/R site (*red sphere*) is located at the intracellular mouth of channel pore (M3–M7, *blue*) (*B*). The exon 4 encoded amino acid in the first intracellular loop (*green sticks*) (*C*). *D*, the raw recording traces of Tmem63b (*QL*) currents responded to hypotonic stimuli. The cells were holding at 0 mV and recorded in whole-cell configuration by ramp protocol from −80 mV to 80 mV, 100-ms duration, 1 Hz. *E*, *left*, hypotonic solution gradually induced Tmem63b (*QL*) currents (measured at −70 mV). *Right*, I-V curves at the indicated time during hypotonic (*red*) to isotonic (*blue*) solutions. *F*, the averaged I-V curves after replacing Na^+^ to Ca^2+^ in hypotonic perfusion solution. *G*, the reversal potential of Ca^2+^ measured by zero-current on I-V plots. QL (−10.4 ± 4.9, *n* = 13), QS (−10.1 ± 7.6, *n* = 9), RL (−23.4 ± 12.6, *n* = 7), RS (−20.3 ± 12.4, *n* = 8). Data are shown as mean ± S.D. (*error bars*). *p < 0.05; one-way ANOVA, Tukey's post hoc test. *H*, the Ca^2+^ permeability relative to Na^+^ (*P_Ca_/P_Na_*) was QL (0.64) ∼ QS (0.72) > RL (0.38) ∼ RS (0.45). Data are shown as mean ± S.D. (*error bars*). *p < 0.05; one-way ANOVA, Tukey's post hoc test. *I*, fluorescent emission images of N2a cells transfected with four Tmem63b isoforms, accompanied with the calcium-sensitive reporter GCaMP6f, in response to 170 mOsm/liter hypotonic stimuli. *QL*, the long isoform without editing. *QS*, the short isoform without editing. *RL*, the long isoform with the edited R. *RS*, the short isoform with the edited R. *White arrowhead* marks the responding cells. *Scale bar*, 10 µm. *J*, percentage of cells responding to 170 mOsm/liter hypotonic solutions. Numbers of cells tested and responding (in parentheses) are indicated above the bars. QL (28.8 ± 4.6%, *n* = 4), QS (41.0 ± 3.6%, *n* = 3), RL (16.7 ± 1.5%, *n* = 3), RS (34.0 ± 2.0%, *n* = 3). Data are shown as mean ± S.D. **p < 0.01; ***p < 0.001; *ns*, not significant; one-way ANOVA, Tukey's post hoc test.

Tmem63 family are osmosensitive (or mechanosensitive) cation channels (27, 39). Their plant orthologue OSCA channels are also osmosensitive and mechanosensitive (39, 40, 41). The Cryo-EM structures of OSCA1.1 and OSCA1.2 have revealed protein domains that are responsible for differential channel properties, including mechanosensation, gating, ion selectivity, and dimer formation (40, 42, 43). To understand whether the editing and splicing affect the channel functions, we made a homology structural model for Tmem63b based on the cryo-EM structure of OSCA1.2 (Fig. 8, *A*–C) (42). The Tmem63b Q/R site is located at the intracellular mouth of the channel pore consisting of transmembrane helices M3–M7 (Fig. 8, *A* and *B*). A charged arginine residue at this position might interfere with the conductance of polyvalent cations, such as Ca^2+^. To test this possibility, we examined the Ca^2+^ conductance relative to Na^+^ for these four isoforms of Tmem63b in Neuro2a (N2a) cells (27). Tmem63b-mediated currents were recorded using a ramp protocol in whole-cell configuration by switching extracellular solution from 300 mOsm/liter to 170 mOsm/liter (Fig. 8, *D* and *E*). We recorded the currents using Na-gluconate in both extracellular and pipette solutions; gluconate was used to avoid the contamination from the endogenous Cl^−^ currents (27). The osmolarity was adjusted by addition of mannitol without changing the ionic concentrations. After Tmem63b currents were induced by 170 mOsm/liter Na^+^ solution and reached the amplitudes of ∼600 pA (measured at −70 mV), the extracellular solution was switched to 170 mOsm/liter Ca^2+^ solution. Because most reacting cells burst with continuous exposure to hypotonic solution, we did not try to record the currents at plateau in this experiment (27). The reversal potential in Na^+^ solution was constant for all four isoforms, −2.1 ± 2.1 mV for QL (the long isoform without editing, *n* = 15), −2.8 ± 2.8 mV for QS (the short isoform without editing, *n* = 9), −2.7 ± 2.2 mV for RL (the long isoform with the edited R, *n* = 7), and −2.9 ± 1.7 mV for RS (the short isoform with the edited R, *n* = 8). The reversal potential in Ca^2+^ solution was −10.4 ± 4.9 mV for QL (*n* = 13), −10.1 ± 7.6 mV for QS (*n* = 9), −23.4 ± 12.6 mV for RL (*n* = 7), and −20.3 ± 12.4 mV for RS (*n* = 8) (Fig. 8, *F* and *G*). The calculated Ca^2+^ permeability relative to Na^+^ (*P_Ca_/P_Na_*) was QL (0.64) ∼ QS (0.72) > RL (0.38) ∼ RS (0.45) (Fig. 8*H*). These data thus demonstrated that Q/R editing at the intracellular mouth of Tmem63b channel reduces Ca^2+^ permeability.

### The splicing and editing regulate hypoosmolarity-induced Ca^2+^ influx

The exon 4 of *Tmem63b* encoded amino acids in the first intracellular loop 1 between M0 and M1 (Fig. 8, *A* and *C*). In *At*OSCA1.2, the intracellular segment intracellular loop 2 paralleling plasma membrane is predicted to be involved in mechanosensation (42, 43). We recently demonstrated that Tmem63s are osmosensitive cation channels activated by hypotonic stress and mediate extracellular Ca^2+^ influx (27). We have established that the most sensitive measurement for the osmosensitivity was the percentage of cells responding to hypotonic stress (27). Interestingly, our measure of osmosensitivity is correlated with the whole-cell currents induced by negative pressure, a direct measure of mechanosensitivity in Tmem63 family proteins (39), indicating that the osmosensitivity reflects the mechanosensitivity. Therefore, we examined the osmosensitivity of the four isoforms of Tmem63b using this established method. In brief, we expressed Tmem63b isoforms accompanied with the calcium reporter GCaMP6f (Tmem63b-P2A-GCaMP6f) in N2a cells (27). GCaMP6f fluorescence was monitored after switching the extracellular osmolarity from 300 mOsm/liter to 170 mOsm/liter. The [Ca^2+^]*_i_* elevation was detected in cells expressing Tmem63b (Fig. 8*I*), but the ratios of responsive cells varied among four isoforms (Fig. 8*J*). Control cells that do not express Tmem63b failed to respond to hypotonic stimuli (Fig. 8*I*; 1/199). Hypoosmolarity-induced Ca^2+^ influx occurred more frequently in cells expressing the short form of Tmem63b (41% of QS and 34% of RS transfected cells) than in cells expressing the long forms (29% of QL and 17% of RL), indicating that exclusion of exon 4 enhanced the osmosensitivity of Tmem63b channel (Fig. 8*J*). These data also showed that Q-form Tmem63bs appeared to be more sensitive to osmolarity change than R-forms. Thus, the alternative splicing of exon 4 and the Q/R editing in Tmem63b coordinately regulate Ca^2+^ influx induced by hypoosmolarity.

## Discussion

In this study, we have identified a coupling between the A-to-I editing at the Q/R site of exon 20 and the alternative splicing of exon 4 in *Tmem63b* pre-mRNAs in the mouse brain. Our study reveals that it displays the following properties. First, the occurrence sites for above post-transcriptional modifications are remote. Second, the alternative RNA splicing plays a dominant role.

In our observation, about 60% of *Tmem63b* mRNAs are edited at the Q/R site in the adult mouse brain, consistent with a previous study (44). The finding that this RNA editing event is brain-specific is also consistent with those reported recently (1, 45, 46). Furthermore, we demonstrate that the editing efficiencies vary in different brain regions at distinct development stages. Lastly, we show that the editing relies on Adar2, in line with previous observations (1, 46), and that the ECS is localized at the proximal 5′ end of intron 20.

We have identified an alternative splicing of exon 4 in brain-originated *Tmem63b* mRNAs. The short *Tmem63b* mRNA isoform lacking exon 4 is the major form in the brain and accounts for about 80% of the total *Tmem63b* mRNAs. The splicing efficiency is constant in different brain regions and relatively stable at development stages. Like the editing, the alternative splicing also occurs mainly in the brain. Although the short isoform accounts for 2% of the total *Tmem63b* mRNAs in the heart, it is not observed in other tissues.

The Q/R editing and alternative splicing events in *Tmem63b* are coupled, in which the alternative splicing of exon 4 plays a dominant role. It has been shown that the exonic A-to-I editing and the splicing of nearby downstream introns often affect each other (22, 23, 24, 25, 26). This could well be explained by interference between the editing machinery and the spliceosomes that may simultaneously act on the adjacent loci on the pre-mRNA. To our knowledge, the interplay between an A-to-I editing and a distant alternative splicing has not been reported before. Our mechanistic analysis reveals that the Q/R editing of *Tmem63b* requires Adar2 binding to the hairpin structure formed by ECS and the editing region. Genetic deletion of Adar2 abolishes the Q/R editing, and mutations on the ECS impair the editing activity (Fig. 3, *C*–*E* and Fig. 4). We suspect that the splicing of exon 4 may regulate the stability of hairpin structure around the editing region or affect Adar2 capability in binding to the structure. Recent evidence has shown that Pin1 promotes the activity of Adar2 and increases the editing efficiencies (47). WWP2 and AIPM2 regulate the degradation of Adar2 to affect RNA editing (1, 47). The splicing factor SRSF9 downregulates Adar2-mediated RNA editing (46, 48). Interestingly, SRSF9 inhibits the *Tmem63b* Q/R editing in the mouse brain (46). Thus, it is possible that the splicing of exon 4 regulates the Q/R editing through some of the above factors.

We have demonstrated that the exon 4 alternative splicing and the Q/R editing regulate the osmosensitivity of Tmem63b. The Q/R site is located at the inner mouth of the Tmem63b channel pore (Fig. 8, *A* and *B*). A positively charged residue at this site reduces the permeability of divalent cation Ca^2+^ (Fig. 8*H*). A similar situation happens in α-amino-3-hydroxy-5-methyl-4-isoxazolepropionic acid receptor GluA2 and kainate receptors GluK1 and GluK2, where the Q/R editing occurs at the center of the channel pore and dictates the Ca^2+^ permeability (4, 49). The splicing site is located in intracellular loop 1 (Fig. 8, *A* and *C*). The structure studies on OSCA channels suggest that the α-helices in intracellular loop 2 paralleling membrane may involve mechanodetection and channel gating (42, 43). Our data demonstrated that the short-form Tmem63s are more sensitive to osmotic changes than long forms. The inclusion of exon 4 may interfere with the α-helices in intracellular loop 2 to change the mechanosensitivity of the channel or regulate osmosensitivity through undetermined mechanisms. It is very likely that the overall Ca^2+^ influx is determined by the Ca^2+^ permeability of the Q/R site combined with the mechanosensitivity of splicing sequences; thus, the apparent osmosensitivity reading by Ca^2+^ influx is QS > QL ∼ RS > RL. Future studies are required to dissect physiological functions of Tmem63b isoforms in the brain.

## Experimental procedures

### Mice

All animal studies were approved by the Institutional Animal Care and Use Committee of Model Animal Research Center of Nanjing University and performed in accordance with guidelines for humane treatment of animals. All animals were housed in 12 h of light and 12 h of dark cycles at 25 ± 1 °C, with water and food obtained *ad libitum*. The *Adar2*^*fl*/+^ mouse line with *loxP* site flanked exons 6–8 was generated through the bacterial artificial chromosome homologous recombination in embryonic stem cells at GemPharmatech (Nanjing). The founder mice were backcrossed with C57BL/6J mice for over five generations followed by breeding with *Nestin-Cre* mice (31) to obtain the brain-specific *Adar2* knockout mice (*Adar2^fl/fl^*; *Nestin-Cre*). Genotypes were determined by PCR with the following primers: 5′-AGTCATTCCTCCTAGCCTTTT-3′ (forward) and 5′-TTATCACCTTGGCATCTTTG-3′ (reverse) to confirm the presence of the *loxP* site, 5′-ATTTGCCTGCATTACCGGTC-3′ (forward) and 5′-ATCAACGTTTTCTTTTCGG-3′ (reverse) for the presence of *Cre*.

### Cloning of Tmem63b, Adar1, and Adar2 from mouse brains

The full-length coding sequences of *Tmem63b* (NM_198167.3), *Adar1* (*Adar*, NM_019655.3), and *Adar2* (*Adarb1*, NM_001024837.2) were amplified from mouse brain cDNAs, using specific primers (Table S4), by PrimeSTAR® HS DNA Polymerase (Takara Bio, R01A) and subcloned into pCAGGS vectors by Ligation-Free Cloning Kit (abm, E001) (50). For measuring the cytoplasmic calcium concentration, the free calcium indicator GCaMP6f was fused to the C-terminal of Tmem63b through a P2A linker (Tmem63b-P2A-GCaMP6f), leading to the separate expression of Tmem63b and GCaMP6f (27).

### Generation of minigene constructs

In the study of A-to-I editing enzyme, the editing segment of *Tmem63b* and the *Htr2c* minigene with 211 bp spanning exon 5 and intron 5 were obtained from mouse genomic DNAs by specific primers (Table S4). The nucleotide in the “B” editing site of *Htr2c* was mutated from the unedited A to the edited G. To explore the ECS, a series of mutations on the editing segment and ECS were obtained through overlapping PCR and corresponding primers (Table S4). In the study of the coupling between alternative splicing of exon 4 and A-to-I editing at the Q/R site, the splicing segments (exon 3∼5) amplified from mouse genomic DNAs and brain cDNAs were fused to the editing segment (LiQi, LQi and SQi) by overlapping PCR and corresponding primers (Table S4). The mutations on SpMs and NvMs were made on the WT LiQi minigene. The intronic segments spliced out from long- or short-form *Tmem63b*, including I3, I4, and I3E4I4, were obtained from mouse genomic DNAs by specific primers (Table S4). These minigenes were subcloned into pCAGGS vectors by Ligation-Free Cloning Kit (abm, E001).

### Cell culture and transfection

HEK 293 cells were cultured in DMEM with 10% FBS (Gibco). The minigenes were transfected into cells using lipofectamine 2000 according to the manufacturer's instruction. The cells were collected for analysis 24–48 h after transfection. CGNs were acutely dissected and cultured following the protocols reported in previous studies (51). In brief, CGNs were prepared from cerebellums of WT mice at P6-8 and plated at a density of 5 × 10^5^ cells/cm^2^ in 10-cm dishes. The cells were cultured in Basal Medium Eagle–based medium containing 10% FBS, 22 mm KCl, 2% B27, 2 mm l-glutamine, and 1% penicillin-streptomycin (all from Gibco) for 2 days. At DIV 2, primary cultured neurons were transfected with *Tmem63b* minigenes using lipofectamine 2000, and the medium was replaced with MEM-based medium containing 5 mg/ml d-glucose (Sigma-Aldrich), 1% ITS (insulin-transferrin-sodium selenite) (Sigma-Aldrich), 2 mm l-glutamine, and 1% penicillin-streptomycin. CGNs were collected for analysis 48–72 h after transfection.

### RNA isolation and quantitative real-time PCR

For cloning and quantification of mRNA isoforms of *Tmem63*b in tissues, total RNA samples from mouse tissues were extracted by TRIzol (Invitrogen) and reverse-transcribed into cDNAs using HiScript® 1st Strand cDNA Synthesis Kit (Vazyme Biotech, R111) and oligo(dT)18 primer. For cultured cells transfected with *Tmem63b* or *Htr2c* minigenes, the extracted total RNAs were digested by RNase-free DNaseI (Life Technologies, AM2238) at 37 °C for 1 h to remove the endogenous genomic and transfected DNAs. Then the RNAs were reverse-transcribed into cDNAs by specific reverse transcription primer, 5′-GAATGCTCGTCAAGAAGAC-3′ (RT_IRES_), using HiScript® 1st Strand cDNA Synthesis Kit (Vazyme, R111). Only the RNAs from exogenous constructs could be reverse-transcribed. The RT (minus) controls of each experiment were carried out to assess the amount of DNA contaminations in RNA preparations.

For quantitative real-time PCR, the RNA samples from mouse brains were reverse-transcribed into cDNAs using HiScript Q RT SuperMix for qPCR (+gDNA wiper) Kit (Vazyme Biotech, R123). cDNAs of different *Tmem63b* isoforms were quantified by Applied Biosystems StepOnePlus Real-Time PCR system (Life Technologies) using AceQ® qPCR SYBR® Green Master (High ROX Premixed) (Vazyme Biotech, Q141) reagents with specific primer pairs: exon 3-4-5, 5′-ACAGATGCAGACAGGCTTC-3′ and 5′-TTGGTCAAAGTCGACAGAGC-3′; exon 3-5, 5′-TGACAGATGCAGACAGTGTG-3′ and 5′-TTGGTCAAAGTCGACAGAGC-3′; exon 5, 5′-ATCAGCTATGCATGGGGAC-3′ and 5′-CCAGGAACAGAAGCCATTG-3′; and *Gapdh*, 5′-TGAACGGGAAGCTCACTGG-3′ and 5′-TCCACCACCCTGTTGCTGTA-3′.

### Quantification of RNA editing

For quantification of Q/R editing in mouse tissues, the genomic sequences spanning the editing site (311 bp) were amplified by primers EDIFw1 (forward) and EDIRe1 (reverse). The cDNA sequences spanning the Q/R site (327 bp) were amplified by primers EDIFw2 (forward) and EDIRe2 (reverse). In the study of A-to-I editing enzyme and ECS in HEK293 cells, the cDNA sequences of transfected *Tmem63b* minigene (403 bp) were amplified by primers pCAGFw (forward) and EDIRe2 (reverse). The cDNA sequence of transfected *Htr2c* minigene (340 bp) was amplified by primers pCAGFw (forward) and pCAGRe (reverse). In the experiment to study the coupling between alternative splicing of exon 4 and Q/R editing at exon 20 in mouse brain cDNAs, the primers LFw (forward) and CRe (reverse) were designed to amplify the long-form (2255 bp) *Tmem63b*, whereas the short-form (2246 bp) *Tmem63b* fragments were amplified by SFw (forward) and CRe (reverse). In the experiment to explore the regulation of splicing to editing in CGNs, the cDNA sequences of transfected *Tmem63b* minigene were amplified by following primers: pCAGFw and EDIRe2 for the study of *trans* regulation, resulting in the PCR a product of 403 bp; and E5Fw (forward) and EDIRe2 (reverse) for the study of *cis* regulation, resulting in the PCR a product of 334 bp. The primers used in above amplification were shown in Table S4.

For *Tmem63b*, the amplified fragments were incubated with BsaWI endonuclease at 60 °C for 1 h and resolved by agarose gel electrophoresis. For *Htr2c*, the amplified fragments were incubated with BtsI endonuclease at 55 °C for 1 h and resolved by agarose gel electrophoresis. The gels were scanned by Gel Imaging System (Tanon). The abundance of each band was analyzed by ImageJ software (National Institutes of Health). The efficiency of editing was displayed by the ratio of integrated densities of BsaWI enzymed bands to control bands. In analysis of ECS, because the BsaWI recognition site is disrupted when the mutations are nearby Q/R site, the editing efficiencies of these mutants were quantified by measuring the ratio of peak heights obtained from sequencing chromatograms.

### Quantification of mRNA splicing isoforms

A pair of primers with forward (SPFw) and reverse (SPRe) were designed to amplify the cDNA sequence near the splicing region (Table S4). *Tmem63b* cDNAs with (long-form) or without (short-from) exon 4 were amplified into different lengths (223 bp and 184 bp), followed by agarose gel electrophoresis. The long (309 bp) and short (270 bp) isoforms from LiQi minigenes were amplified by pCAGFw (forward) and E5Re (reverse), followed by agarose gel electrophoresis (Table S4). The gels were scanned by Gel Imaging System (Tanon). The abundance of each band was analyzed by ImageJ software (National Institutes of Health). The values were normalized according to bands size difference. The efficiency of exon 4 alternative splicing was displayed by the ratio of integrated densities of short form bands to total.

### Cytoplasmic Ca^2+^ measurements

The cytoplasmic calcium concentration was monitored by free calcium indicator GCaMP6f (27). Tmem63b-P2A-GCaMP6f vectors were transfected into N2a cells mounted on the coverslip. GCaMP6f vector was used as a control. 40 h after transfection, the cells were perfused with isotonic extracellular solution (in mm): 65 NaCl, 5 KCl, 1 CaCl_2_, 1 MgCl_2_, and 10 HEPES (pH 7.4 adjusted with NaOH; 300 mOsm/liter adjusted with mannitol). The isotonic solution was exchanged to 170 mOsm/liter hypotonic solution without changing the ionic concentrations by a peristaltic pump (BT100-2J, Longer Precision Pump Co., Ltd., Hebei, China) at a constant speed. The osmolarity was measured by a vapor pressure osmometer (Wescor Vapro Model 5600). The cytoplasmic calcium fluorescence was recorded at 1 Hz for 10 min by laser scanning confocal microscope (Leica Microsystems, TCS SP2) at room temperature (24 ± 2 °C) using 488-nm illumination. The change of fluorescence was normalized by the ratio of real-time intensity (Ft) relative to the initial value (F0). The cells with Ft/F0 > 1.5 were considered as positive cells responding to osmolarity changes.

### N2a cell electrophysiology

Whole-cell path clamp recordings were performed on transfected N2a cells under ramp protocol. The recording electrodes had the resistance of 8∼10 MΩ when filled with the pipette solution composed of (in mm) 80 Na-gluconate, 130 mannitol, and 10 HEPES (pH 7.4 with NaOH, 300 mOsm/liter). The cells were perfused with isotonic extracellular solution containing (in mm) 80 Na-gluconate, 1 Ca-gluconate, 10 HEPES, and 130 mannitol (pH 7.4 with NaOH, 300 mOsm/liter). The hypotonic extracellular solution without changing the ionic concentration contains (in mm) 80 Na-gluconate, 1 Ca-gluconate, and 10 HEPES (pH 7.4 with NaOH, 170 mOsm/liter). For measuring the Ca^2+^ permeability, the Na^+^-rich hypotonic extracellular solution was changed to Ca^2+^-rich solution containing (in mm) 40 Ca-gluconate, 40 mannitol, and 10 HEPES (pH 7.4 with Ca(OH)_2_, 170 mOsm/liter). The currents were collected using an Axopatch 200B amplifier and Digidata 1550 digitizer (Molecular Devices) at the sampling rate of 10 kHz and were low-pass filtered at 1 kHz. The current data were analyzed using pClamp 10 software. The cells were holding at 0 mV before application of 100-ms ramp from −80 mV to 80 mV every 1 s. The cells with a membrane resistance below 800 MΩ or series resistance above 10 MΩ were discarded. The Ca^2+^ permeability relative to Na^+^ (P_Ca_/P_Na_) was calculated as P_Ca_/P_Na_ = [Na^+^]*_i_*exp(ΔV_rev_*F/RT*) (1+exp(ΔVrev*F/RT*)) / 4[Ca^2+^]*_o_*, where the ΔVrev is measured the shift in reversal potential, [Na^+^]*_i_* is the intracellular Na^+^ concentration, and [Ca^2+^]*_o_* is the concentration of the extracellular substituting Ca^2+^. The reversal potential was measured in 7–13 cells for each isoform.

### RNA secondary structure prediction

The secondary structures of RNA around the editing site and the ECS were predicated by RNAstructure software (Version 6.0.1). The sequence 200 bases downstream and upstream of the editing site was imported for analysis. The lowest free energy structure and a set of low free energy structures were predicted according to following restrictions on parameters: Maximum % Energy Difference, Maximum Number of Structures, and Window Size. The parameters were set as previously reported (33). The Maximum % Energy Difference was 10%. The Maximum Number of Structures was 20. The Window Size was 3.

### Homology modeling

Protein sequence of mouse Tmem63b was aligned to *At*OSCA1.2, the plant homolog of Tmem63b with Cryo-EM structure solved (42, 43). The homology modeling of mouse Tmem63b was obtained using SWISS-MODEL server (53) with the template of *At*OSCA1.2 (PDB ID: 6mjv). The model was depicted by PyMol program.

### Statistical analysis

All data are presented as mean ± standard deviation (S.D.) in at least three independent experiments. Statistical analyses were performed using GraphPad Prism version 6.0c software and analyzed using one-way analysis of variance (ANOVA) or unpaired *t* test if not otherwise stated. *p* values less than 0.05 were considered statistically significant. *p < 0.05; **p < 0.01; ***p < 0.001; ****p < 0.0001. *p* ≥ 0.05 was denoted as “*ns.*”

## Data availability

All data supporting our conclusions are contained within this article and in the supporting information.
