# Supplementary material for: Distant coupling between RNA editing and alternative splicing of the osmosensitive cation channel Tmem63b
Source: J Biol Chem. 2021 Jan 13;295(52):18199–212. doi: 10.1074/jbc.RA120.016049 (PMC7939439; doi:10.1074/jbc.RA120.016049)
Supplement: Supplementary file 1 [file mmc1.pdf]

SUPPORTING DATA

Distant Coupling between RNA Editing and Alternative Splicing of the Osmosensitive Cation Channel Tmem63b

Dan Wu, Yan-Yu Zang, Yong-Yun Shi, Chang Ye, Wen-Min Cai, Xiao-Hui Tang, Liyun Zhao, Yong Liu, Zhenji Gan, Gui-quan Chen,Yun Xu, Jian-Jun Yang, Yun Stone Shi

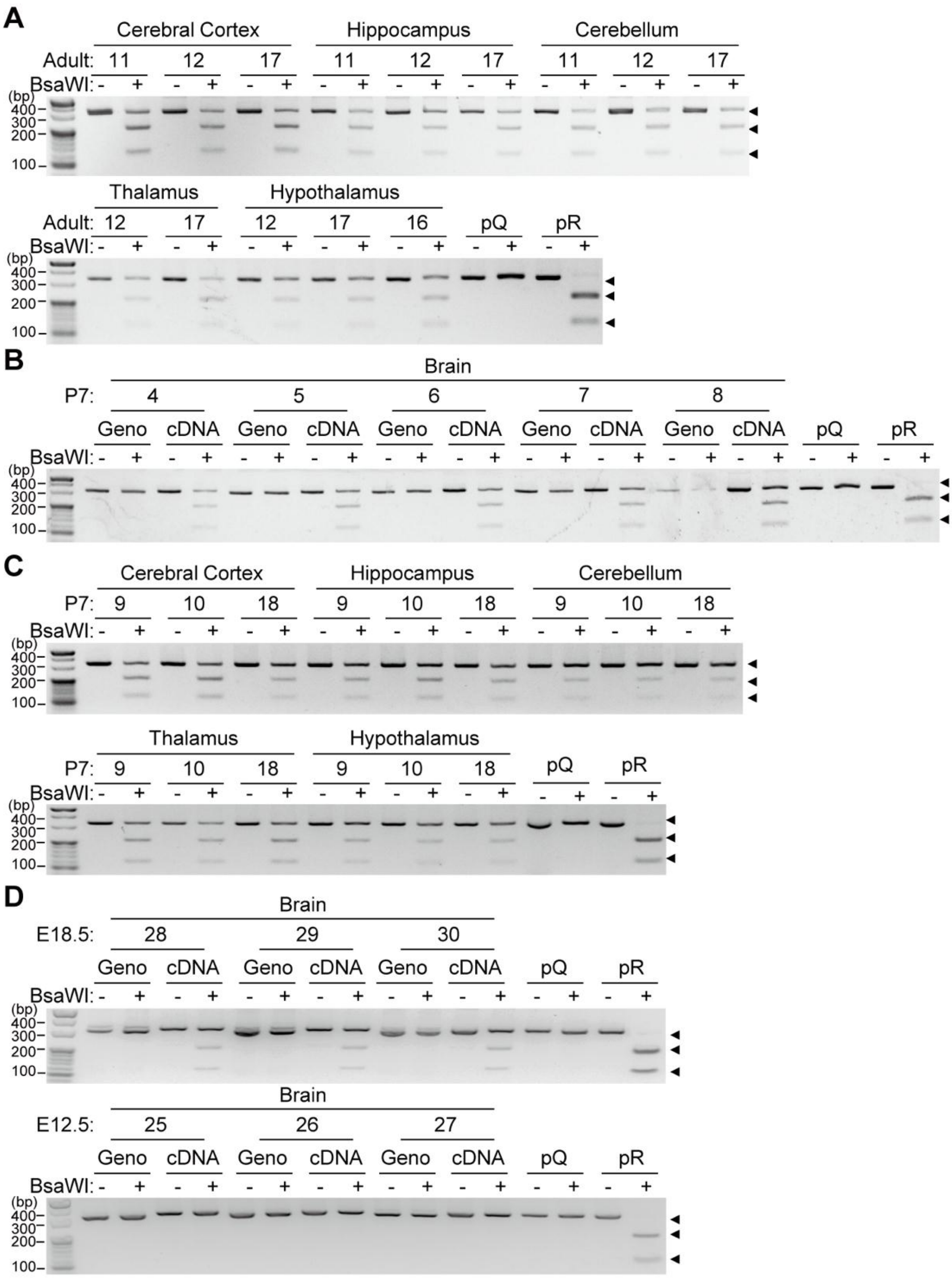

Figure S1. Q/R editing of *Tmem63b* in different brain regions and ages. *A*, Q/R editing of *Tmem63b* in different brain regions from three adult mice with codes labelled above. *B*, Q/R editing of *Tmem63b* in brains from P7 mice with codes labelled above. *C*, Q/R editing of *Tmem63b* in different brain regions from P7 mice with codes labelled above. *D*, Q/R editing of *Tmem63b* in brains of embryonic (E12.5, E18.5) mice with codes labelled above.

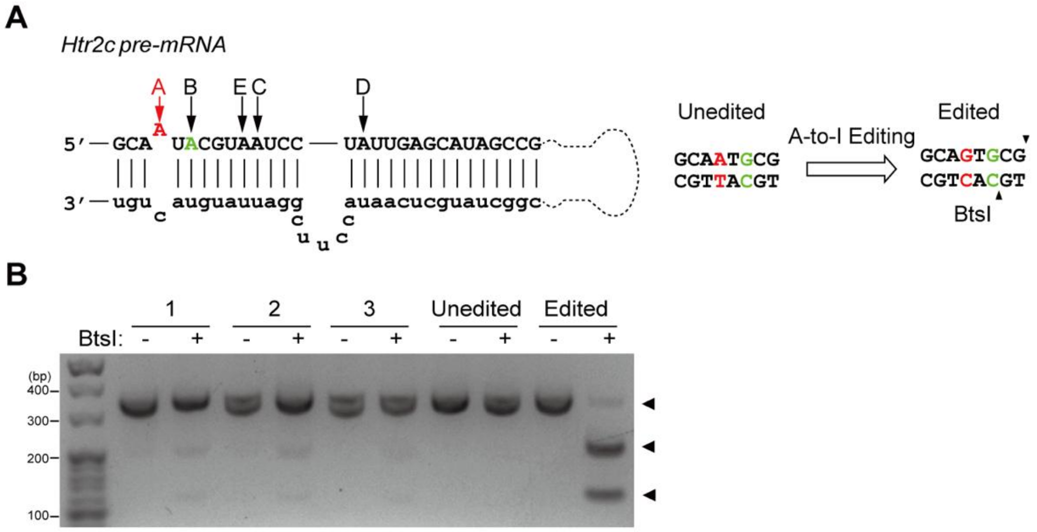

Figure S2. The A-to-I editing of 'A' site in *Htr2c* minigene co-transfected with Adar1. *A*, schematic representation of dsRNA hairpin structure formed by exon 5 and intron 5. The reported editing sites, 'A' to 'E', were marked in arrows. In order to evaluate the editing efficiency of 'A' site (red) individually, the adjacent 'B' site (green) was mutated to the edited G. In this mutant, BtsI endonuclease recognizing site (right panel) was introduced after A-to-I editing at 'A' site. *B*, the A-to-I editing of 'A' site in *Htr2c* minigene co-transfected with *Adar1* in HEK 293 cells. The fragment (340bp) amplified by specific primers were partially digested into two bands (122bp and 218bp) by BtsI. The experiments were repeated for three times.

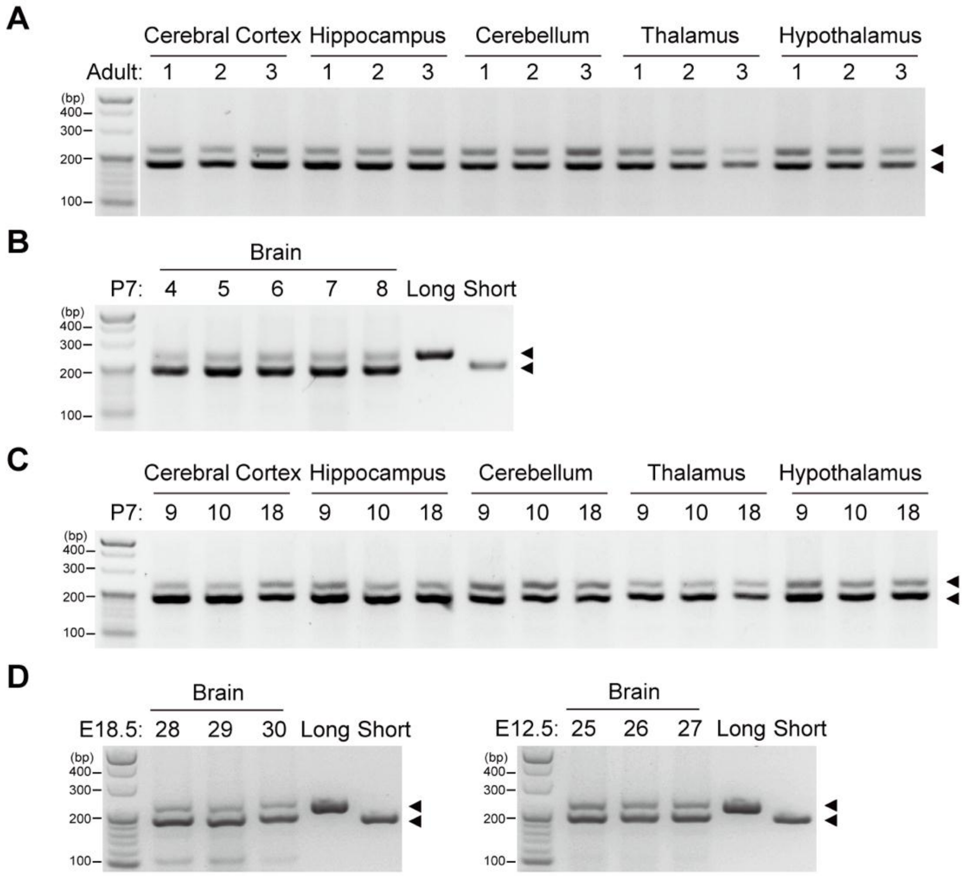

Figure S3. Alternative splicing of *Tmem63b* exon 4 in different brain regions and ages. *A,* alternative splicing of *Tmem63b* exon 4 in different brain regions from three adult mice with codes labelled above. *B,* alternative splicing of *Tmem63b* exon 4 in brains of P7 mice with codes labelled above. *C,* alternative splicing of *Tmem63b* exon 4 in different brain regions from three P7 mice with codes labelled above. *D,* alternative splicing of *Tmem63b* exon 4 in brains of embryonic (E12.5, E18.5) mice with codes labelled above.

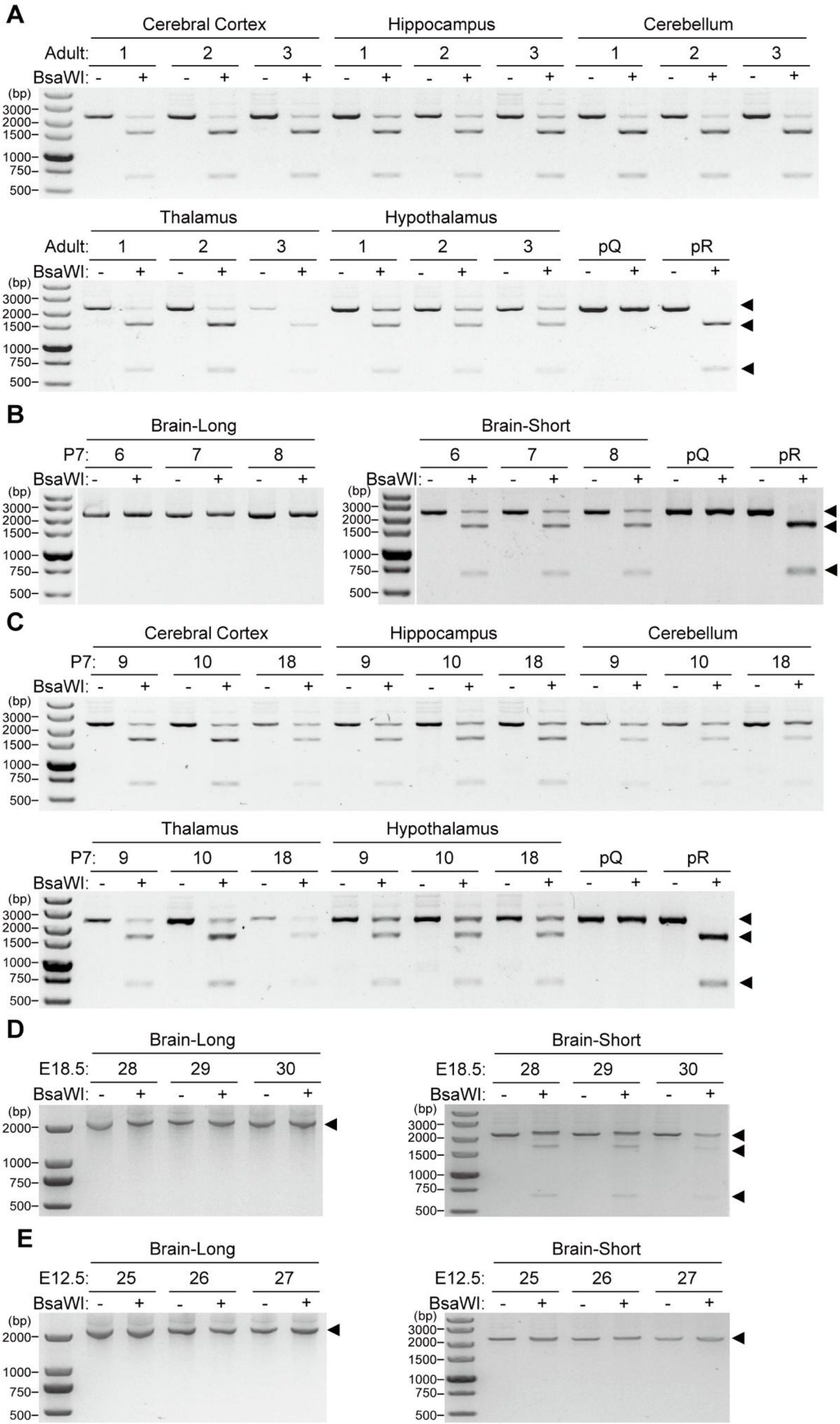
A
Cerebral Cortex
Hippocampus
Cerebellum
Adult: 1 2 3 1 2 3 1 2 3
BsaWI: - + - + - + - + - + - + - + - + - +
(bp)
3000–
2000–
1500–
1000–
750–
500–
Thalamus
Hypothalamus
Adult: 1 2 3 1 2 3 pQ pR
BsaWI: - + - + - + - + - + - + - + - +
B
Brain-Long
P7: 6 7 8
Brain-Short
6 7 8 pQ pR
C
Cerebral Cortex
Hippocampus
Cerebellum
P7: 9 10 18 9 10 18 9 10 18
Thalamus
Hypothalamus
P7: 9 10 18 9 10 18 pQ pR
D
Brain-Long
E18.5: 28 29 30
Brain-Short
E18.5: 28 29 30
E
Brain-Long
E12.5: 25 26 27
Brain-Short
E12.5: 25 26 27

Figure S4. The coupling between exon 4 alternative splicing and Q/R editing of *Tmem63b* in different brain regions and ages. *A*, Q/R editing of short form *Tmem63b* in different brain regions from three adult mice with codes labelled above. *B*, Q/R editing of long form (left) and short form (right) *Tmem63b* in brains of p7 mice with codes labelled above. *C*, Q/R editing of short form *Tmem63b* in brain regions from three P7 mice with codes labelled above. *D and E*, Q/R editing of long form (left) and short form (right) *Tmem63b* in brains of embryonic (E12.5, E18.5) mice with codes labelled above.

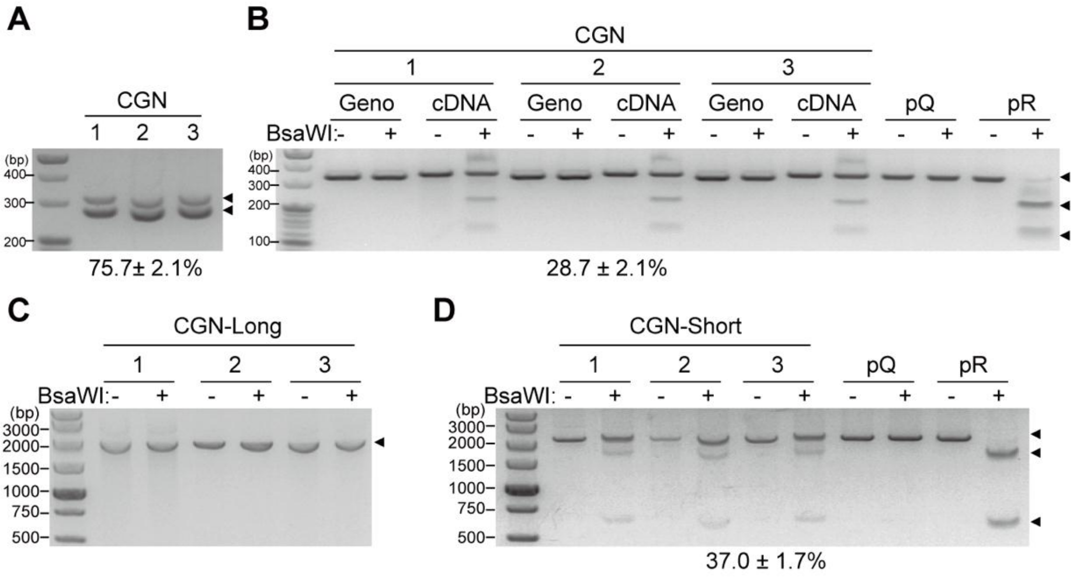

Figure S5. Alternative splicing (A) and Q/R editing (B) of endogenous *Tmem63b* in primary cultured CGNs. *C* and *D*, Q/R editing of long form and short form *Tmem63b* in CGNs. The experiments were repeated in three independent culturing of CGNs.

Table S1. Q/R Editing in different brain regions of P7 and adult mice (Fig 2*D*)

Editing (%)	Cx	Hi	Cb	Th	Hy
P7	53.0 ±1.4, n=3	37.3 ±2.3, n=3	19.7 ±0.6, n=3	52.0 ±1.0, n=3	30.0 ±1.7, n=3
Adult	64.8 ±2.2, n=5	53.6 ±3.0, n=5	58.0 ±1.2, n=5	65.0 ±2.9, n=4	36.5 ±4.4, n=6

Table S2. Alternative splicing in different brain regions of P7 and adult mice (Fig 5*D*)

Editing (%)	Cx	Hi	Cb	Th	Hy
P7	80.5 ±0.7, n=3	76.0 ±1.7, n=3	65.0 ±1.7, n=3	76.0 ±0.0, n=3	69.0 ±1.7, n=3
Adult	75.3 ±3.1, n=6	73.7 ±3.4, n=6	68.3 ±3.9, n=6	76.0 ±6.5, n=4	67.4 ±3.1, n=5

Table S3. Q/R Editing of short form *Tmem63b* in different brain regions of P7 and adult mice (Fig 6*F*)

Editing (%)	Cx	Hi	Cb	Th	Hy
P7	74.5 ±0.7, n=3	60.7 ±1.2, n=3	42.5 ±2.1, n=3	78.0 ±0.0, n=3	52.3 ±1.5, n=3
Adult	84.6 ±2.8, n=5	74.0 ±2.0, n=5	85.7 ±1.5, n=6	85.3 ±2.5, n=3	61.3 ±3.3, n=4

TableS4. Primers for cloning and amplifications

Gene	Primer	Sequence (5'-3')
Tmem63b	Tmem63b-Fw	5’-ATTCGCGGCCGCTAGCATGCTGCCGTTCTTGCTG-3’
	Tmem63b-Re	5’-GAAGCTTGAGCTCGAGTTACTGGTGAATCTCATTC-3’
Adar1	Adar1-Fw	5’-ATTCGCGGCCGCTAGCATGTCTCAAGGGTTCAGG-3’
	Adar1-Re	5’-GAAGCTTGAGCTCGAGTCAGTCATTGGGTACTGGAC-3’
Adar2	Adar2-Fw	5’-ATTCGCGGCCGCTAGCATGGATATAGAAGATGAAGAG-3’
	Adar2-Re	5’-GAAGCTTGAGCTCGAGTCAGGGAGTGAAGGAGAAC-3’
Editing segment	Ex19-Fw	5’-ATTCGCGGCCGCTAGCGTGTGTGTTCCTGCCCGAC-3’
	Ex21-Re	5’-GAAGCTTGAGCTCGAGCCGTGCGCATGGTGGAGAAG-3’
Htr2c	Ht(BG)-Ex5-Fw	5’-ATTCGCGGCCGCTAGCGGACCGGTATGTAGCAATGCGTAATC-3’
	Ht-In5-Re	5’-GAAGCTTGAGCTCGAGGACAACCGATCAAACGCAATG-3’
Splicing-editing fusing minigene	Ex3-Fw	5’-ATTCGCGGCCGCTAGCGCCCTGCTGTTCTTATTC-3’
	Ex5-Re(fs)	5’-ATGCGTGACTATTGTCCCTTTGGTCAAAG-3’
	Ex19-Fw(fs)	5’-ACAATAGTCACGCATGTGTGTGTTCCTGCCCGAC-3’
	Ex21-Re	5’-GAAGCTTGAGCTCGAGCCGTGCGCATGGTGGAGAAG-3’
	Ex4(CA)-Fw	5’-GAGAGGGAGCGAGTGGAACAGGAATACAATGTGGGGCATCAG CCCCCTC-3’
	(TC)Ex4-Re	5’-CACTCGCTCCCTCTCCTGCCGGCGAAGCGAGGGGACAGAGAAC AGAAAG-3’
	NovaM-Fw1	5’-CAAACAAGTGACTGGTGGTTGC-3’
	NovaM-Re1	5’-GCAACCACCAGTCACTTGTTTG-3’
	NovaM-Fw2	5’-CTCTGTCGGTGACTGGTC-3’
	NovaM-Re2	5’-GACCAGTCACCGACAGAG-3’
	NovaM-Fw3	5’-CAGGGCCTCAGTACTGCTGCAC-3’
	NovaM-Re3	5’-GTGCAGCAGTACTGAGGCCCTG-3’
I3	TM63B-In3-Fw	5’-CAAAGAATTCGCGGCCGCGTAAGAAAGAGCCCGAGCCTG-3’
	TM63B-In3-Re	5’-ATCTAGAGTCGCGGCCGCCTGGGGACAGAGAACAGAAAG-3’
I4	TM63B-In4-Fw	5’-CAAAGAATTCGCGGCCGCGTATGTGGGGCATCAGCCCCCTC-3’
	TM63B-In4-Re	5’-ATCTAGAGTCGCGGCCGCCTGGGGGGTGGGGAGACAG-3’
I3E4I4	TM63B-In3-Fw	5’-CAAAGAATTCGCGGCCGCGTAAGAAAGAGCCCGAGCCTG-3’
	TM63B-In4-Re	5’-ATCTAGAGTCGCGGCCGCCTGGGGGGTGGGGAGACAG-3’
Mut1	Mut1-Fw	5’-CTGGGCCCTGCTCGGGGGAAC-3’
	Mut1-Re	5’-GTTCCCCCGAGCAGGGCCCAGGTGCCTACCGAAGGGCAC-3’
Mut2	Mut2-Fw	5’-CACCGCCGCGCCGGGTCCTG-3’
	Mut2-Re	5’-CAGGACCCGGCGCGGCGGTG-3’
Mut3	Mut3-Fw	5’-CGCCGCGACCTGGGCCCTG-3’
	Mut3-Re	5’-CAGGGCCCAGGTCGCGGCG-3’
Mut4	Mut4-Fw	5’-CACCGCCGCGCTGGGACCTG-3’
	Mut4-Re	5’-CAGGTCCCAGCGCGGCGGTG-3’
Mut5	Mut5-Fw	5’-CACCGCCGCGGCGGGACCTG-3’
	Mut5-Re	5’-CAGGTCCCGCCGCGGCGGTG-3’

Mut6	Mut6-Fw	5’-CACCGGCGCGCCGGGACCTG-3’
	Mut6-Re	5’-CAGGTCCCGGCGCGCCGGTG-3’
Mut7	Mut7-Fw	5’-CACCGGCGCGGTGGGTCCTG-3’
	Mut7-Re	5’-CAGGACCCACCGCGCCGGTG-3’
Mut8	Mut8-Fw	5’-GGACCTTCCGCCTGCTCG-3’
	Mut8-Re	5’-CGAGCAGGCGGAAGGTCC-3’
Mut9	Mut9-Fw	5’-GTGCCCTTCGGTTTCCACC-3’
	Mut9-Re	5’-GGTGGAAACCGAAGGGCAC-3’
Mut10	Mut10-Fw	5’-CTACGAGATCCAGTTTGGC-3’
	Mut10-Re	5’-GCCAAACTGGATCTCGTAG-3’
Mut11	Mut11-Fw	5’-CTACGAGTTGCAGTTTGGC-3’
	Mut11-Re	5’-GCCAAACTGCAACTCGTAG-3’
Mut12	Mut12-Fw	5’-CTACGAGTTCCACTTTGGC-3’
	Mut12-Re	5’-GCCAAAGTGGAACTCGTAG-3’

Primer	Sequence (5'-3')
EDIFw1	5’-ACATTAGGTCCCTCACTC-3’
EDIRe1	5’-TGCCTTACTAGAGTGGAG-3’
EDIFw2	5’-CTGGCTGAAGCAGCTATTCG-3’
EDIRe2	5’-TACCAGGTGCTTCAGCAGC-3’
SPFw	5’-TCTGGACTTCATGTGCTTTC-3’
SPRe	5’-TTGGTCAAAGTCGACAGAGC-3’
LFw	5’-AGAGGGAGCGAGTGGAACAG-3’
SFw	5’-TGACAGATGCAGACAGTGTG-3’
CRe	5’-CCGCTCGAGTTACTGGTGAATCTCATTCTC-3’
E5Fw	5’-GACTTTGACCAAAGGGAC-3’
E5Re	5’-GTCCCTTTGGTCAAAGTC-3’
pCAGFw	5’-GCTAACCATGTTCATGCCTTC-3’
pCAGRe	5’-CTGCAGAATTCGAAGCTTG-3’
